# IRAK-M suppresses the activation of microglial NLRP3 inflammasome and GSDMD-mediated pyroptosis through inhibiting IRAK1 phosphorylation during experimental autoimmune encephalomyelitis

**DOI:** 10.1038/s41419-023-05621-6

**Published:** 2023-02-10

**Authors:** Yuanyuan Wang, Shanshan Pei, Zhuhe Liu, Yuewen Ding, Tinglin Qian, Haixia Wen, Ssu-Wei Hsu, Zheyi Zhou, Jun Zhang, Honghao Wang

**Affiliations:** 1 Department of Neurology, Guangzhou First People’s Hospital, School of Medicine, South China University of Technology, 510180 Guangzhou, China; 2 Department of Neurology, Nanfang Hospital, Southern Medical University, 510515 Guangzhou, China; 3 Department of Internal Medicine, University of California at Davis, Davis, CA 95616 USA; 4 Department of Neurology, Hospital of Liuzhou Traditional Chinese Medicine, 545001 Liuzhou, China; 5 Comprehensive Cancer Center, University of California at Davis, Davis, CA 95616 USA

**Keywords:** Neuroimmunology, Autoimmunity

## Abstract

The activation of the NOD-like receptor family pyrin domain-containing protein 3 (NLRP3) inflammasome triggers pyroptosis proinflammatory cell death in experimental autoimmune encephalomyelitis (EAE). However, the underlying mechanisms of the inflammatory processes of microglia in EAE remain unclear. Our previous studies suggested that interleukin-1 receptor-associated kinase (IRAK)-M down-regulates the toll-like receptor 4/interleukin-1 receptor signaling pathway. Here, we used IRAK-M knockout (IRAK-M^−/−^) mice and their microglia to dissect the role of IRAK-M in EAE. We found that deletion of IRAK-M increased the incidence rate and exacerbated the clinical symptoms in EAE mice. We then found that IRAK-M deficiency promoted the activation of microglia, activated NLRP3 inflammasomes, and enhanced GSDMD-mediated pyroptosis in the microglia of EAE. In contrast, over-expression of IRAK-M exerted inhibitory effects on neuroinflammation, NLRP3 activation, and pyroptosis. Moreover, IRAK-M deficiency enhanced the phosphorylation of IRAK1, while IRAK-M over-expression downregulated the level of phosphorylated IRAK1. Finally, we found upregulated binding of IRAK1 and TNF receptor-associated factor 6 (TRAF6) in IRAK-M^−/−^ EAE mice compared to WT mice, which was blocked in AAV^IRAK-M^ EAE mice. Our study reveals a complex signaling network of IRAK-M, which negatively regulates microglial NLRP3 inflammasomes and pyroptosis by inhibiting IRAK1 phosphorylation during EAE. These findings suggest a potential target for the novel therapeutic approaches of multiple sclerosis (MS)/EAE and NLRP3-related inflammatory diseases.

## Introduction

Multiple sclerosis (MS) is an immune-mediated chronic demyelinating disease, which is characterized by inflammatory infiltrates, myelin loss, and axonal damage of the brain and spinal cord. The typical neuropathology of MS includes a progressive demyelinating disorder that predominantly affects the white matter [1, 2]. Experimental autoimmune encephalomyelitis (EAE) shares many clinical and pathological similarities with MS. Studies have revealed that both innate and adaptive immune cells contribute to the pathogenesis and progression of MS. As MS develops, an influx of inflammation-infiltrating cells accumulates mainly in lesions of the white matter in the central nervous system (CNS), characterized by T cells and microglia [3, 4]. The encephalitogenic signaling pathways that account for EAE mainly focus on the toll-like receptor (TLR) 4-mediated immune response [5–7].

Recent evidence has suggested the critical role of microglial paralysis in the pathogenesis of MS [8–10]. The status of classically activated microglia (M1) manages the inflammatory extent during EAE modeling, mainly through promotion of proinflammatory cytokine release and effector T cell activation, while M2 microglia inhibit the proinflammatory process and facilitate tissue repair by promoting the regulatory T cell response and secreting anti-inflammatory cytokines such as interleukin (IL)-10 [11–13]. Furthermore, microglial paralysis obviously suppresses the development of EAE [14, 15]. Altogether, microglia have a pivotal role in the pathogenesis of both MS and EAE. In the past decade, NOD-like receptor family pyrin domain-containing protein 3 (NLRP3) inflammasome activation has been reported to be associated with various CNS diseases, including Alzheimer’s disease, Parkinson’s disease, and epilepsy [16–19]. Previous study on blood samples of patients with relapsing-remitting MS confirmed that the RNA expression levels of NLRP3 and IL-1β are upregulated in the acute phase and that the NLRP3 inflammasome is positively correlated with the response to interferon-β therapy in these patients [20]. It has recently been proposed that the activation of the NLRP3 inflammasome in microglia is induced by the pore-forming protein gasdermin D (GSDMD), which mediates pyroptosis, leading to the formation of membrane pores and cell swelling [21, 22]. Therefore, the microglial NLRP3 inflammasome may influence CNS physiopathology via pyroptosis and act as a proinflammatory amplifier in MS/EAE.

The NLRP3 inflammasome is a cytoplasmic caspase-1 activated protein complex mainly expressed in microglia of the CNS consists of three molecules: NLRP3, pro-caspase-1, and adapter protein apoptosis-associated speck-like protein (ASC) [23, 24]. After sensing a broad range of pathogen-associated molecular patterns and damage‐associated molecular patterns, the molecules mentioned above quickly accumulate to form a large oligomer, which is a crucial sensor of the cytoplasm. [25, 26]. As an extensive inflammatory complex, the activation of the NLRP3 inflammasome elicits caspase-1 autocatalytic activation and subsequently facilitates the maturation and rapid secretion of proinflammatory cytokines, such as IL-1β and IL-18, which trigger a cascade of inflammatory responses through various pathways, including the nuclear factor kappa-B (NF-κB), activator protein 1, and c-Jun N-terminal kinase (JNK) [27, 28], and finally exacerbates EAE through robust recruitment of immune cells in the white matter of MS patients, increasing neurological disability [18, 29–31]. Importantly, active caspase-1 together with caspase-4/5/11 cleave GSDMD to induce “pyroptosis”, a type of inflammatory cell death that releases cytosolic inflammatory cytokines such as IL-1β [22, 32–34]. Indeed, increasing evidences in patients with MS and EAE models have suggested that both the NLRP3 inflammasome activation and pyroptosis in microglia account for the robust inflammation and the deterioration in the progress of this disease [21]. Considering the pathogenic function of microglial NLRP3 inflammasomes and the occurrence of pyroptosis in MS/EAE, the related upstream molecules concerning the inhibition of their activity remain to be investigated.

Numerous studies point to a crucial role of TLR4 signaling stimulation in EAE [5–7, 35]. Downstream signaling following the binding of divergent pathogen ligands to TLR4 involve the formation of MyD88/IRAK4/IRAK1 myddosomes, resulting in IRAK1 auto-phosphorylation and dissociation of IRAK1 from the complex. Immediately after, phosphorylated IRAK1 binds to TRAF6 to activate downstream signaling pathways driving a robust release of proinflammatory cytokines, such as IL-1β, IL-6, IL-23, and tumor necrosis factor (TNF)-α [5, 36, 37]. Interestingly, our previous study demonstrated a protective effect of microglial IRAK-M in alleviating EAE progression in C57BL/6 mice, revealing that IRAK-M significantly suppresses the disease scores and M1 microglia infiltration in lesions of spinal cords [38]. IRAK-M, also termed IRAK3, is an inactive member of the IRAK family, which is mainly expressed on microglia in the CNS [39, 40]. Various studies have provided information about the inhibitory role of IRAK-M in the TLR4 pathway, leading to the prevention of IRAK1 phosphorylation and subsequently suppressing dissociation from the MyD88/IRAK4/IRAK1 complex, preventing the formation of the IRAK1/TRAF6 complex [40, 41]. Considering the rapid activation of NLRP3 inflammasomes by IRAK1, and the important role of NLRP3 inflammasomes in the pathogenesis of EAE, we speculate that IRAK-M may suppress the activation of NLRP3 inflammasomes and pyroptosis in microglia and subsequently attenuate the onset of MS/EAE in an IRAK1-dependent manner. Here, we confirm NLRP3 inflammasome activation and pyroptosis, as well as an enhanced inflammatory response in microglia in the EAE model. Further, IRAK-M deletion was shown to exacerbate the infiltration of activated microglia in the spinal cord and aggravate clinical symptoms in C57BL/6 EAE mice. Taken together, our findings suggest that IRAK-M suppresses the activation of NLRP3 inflammasomes and pyroptosis through the inhibition of IRAK1 phosphorylation and the IRAK1/TRAF6 combination in EAE.

## Materials and methods

### Reagents

MOG_35–55_ peptide (MEVGWYRSPFSRVVHLYRNGK) was purchased from GL Biochem Inc. (Shanghai, China); Complete Freund’s adjuvant (CFA) (#7001, *M. tuberculosis H-37 RA*, 4 mg/ml) was purchased from Chondrex Inc. (Woodinville, WA, USA); and Pertussis toxin (PTX) (#3097) and nigericin (#4312/10) were purchased from Tocris Bioscience (Bristol, UK). IRAK1 polyclonal antibody (#PA5-19855), Goat anti-Mouse IgG, Goat anti-Rabbit IgG, Alexa Fluor 488 (#A-11029), Alexa Fluor 488 (#A-11034), Alexa Fluor 647 (#A-11034), Alexa Fluor 594 (#A-11032), Alexa Fluor 647 (#A-32728), Donkey anti-Goat IgG, Alexa Fluor 488 (#A-11055), phosphate buffered saline (PBS) (#10010049), fetal bovine serum (#10100147), penicillin/streptomycin (#15140122), Dulbecco’s Modified Eagle Medium (DMEM) (#11965092), Pierce™ Rapid Gold BCA Protein Assay Kit (#A53225), Pierce™ Protein A/G Magnetic Beads (#88803), and TRIzol® Reagent (#15596018) were purchased from Thermo Fisher Scientific (Waltham, MA, USA). Human/mouse NLRP3/NALP3 antibody (#MAB7578) was purchased from R&D Systems (MN, USA); ASC antibody (#NBP1-78978) and GSDMDC1 antibody (#NBP2-33422) were purchased from Novus Biologicals (CO, USA); caspase-1 antibody (#sc-56036) was purchased from Santa Cruz Biotechnology (Dallas, Texas, USA); cleaved IL-1β (#83186) was purchased from Cell Signaling Technology (Danvers, USA); Iba1 antibody (#ab5076) and mounting medium with DAPI (#ab104139) were obtained from Abcam (Cambridge, UK); goat anti-rabbit IgG (H+L)-HRP(#FDR007), goat anti-mouse IgG (H+L)-HRP (#FDM007), and RIPA buffer (#FD009, for immunoblot; #FD011, for co-immunoprecipitation) were purchased from FDBIO SCIENCE (Hangzhou, Zhejiang, China); DAB staining solution (#ZLI-9017) was purchased from ZSGB BIO (Beijing, China); hematoxylin and eosin (H&E) staining solution (#BA4027); and Luxol Fast Blue (LFB) staining solution (#BA4357A) were purchased from Baso Inc. (Zhuhai, Guangdong, China); optimum cutting temperature (OCT) compound (#4583) was purchased from Sakura Finetek Inc. (Torrance, CA, USA); normal donkey serum (#SL050), Tris Buffered Saline with Tween® 20 (TBST) (#T1085), and bovine serum albumin (BSA) (#A8020) were purchased from Solarbio Inc. (Beijing, China); Immoblilon PVDF membranes (#ISEQ00010) and Immobilon Western Chemiluminescent HRP Substrate (#WBKLS0100) were purchased from Merck (Darmstadt, Germany); lipopolysaccharide (LPS) (#L2630) was purchased from Sigma-Aldrich (St. Louis, MO, USA); PCR primers were obtained from Sangon Biotech (Shanghai, China); PrimeScript RT Master Mix Kit (#RR036A) and SYBR Premix Ex Taq II Kit (#RR820A) were purchased from Takara Bio Inc. (Shiga, Japan); isoflurane was obtained from RWD Inc. (Shenzhen, China); and the vector adeno-associated virus (AAV) 9-IRAK-M was designed by Hanbio Biotechnology (Shanghai, China).

### Animals

C57BL/6 female mice were purchased from the Experimental Animal Center of Southern Medical University. All of the mice were bred in the Southern Medical University Animal Feeding Center under specific pathogen-free conditions. The environmental conditions were as follows: T 21 ± 1 °C, 50%–60% relative humidity, 12/12-h light/dark cycle. All animal experiments adhered to the ARRIVE guidelines. All of the animal care and experimental procedures were performed in accordance with the NIH Guide for the Care and Use of Laboratory Animals (NIH, revised 1996) and approved by the Laboratory Animal Ethics Committee of Southern Medical University (Guangzhou, China). A total of 212 C57BL/6 mice were enrolled in this study, including 68 IRAK-M^−/−^ mice. Among them, 58 mice were assigned to the CFA group, 154 mice were used to induce the EAE model, and 56 mice were assigned to AAV injection. Animals were randomly assigned to each group, and animal experiments were carried out in a blind manner. Experimenters were blind to the genotype of the animal and group assignment.

### Induction of EAE and model assessment

C57BL/6 (9–11-week-old female) mice of different groups were immunized subcutaneously with 200 μg MOG_35–55_ peptide emulsified in CFA (4 mg/mL *Mycobacterium* tuberculosis). Mice in the control group (CFA group) were induced with only PBS emulsified in the above emulsion. All of the mice received 200 ng PTX injection intraperitoneally on the day of immunization and 2 days later. The clinical score and weight of the mice in each group were blindly evaluated every day until 30 days after immunization. Model assessment was based on the standardized 0–5 grading criteria as follows: 0, no symptoms of disease; 1, a decrease in tail tonicity; 2, hind limb weakness or paresis; 3, complete hind limb paralysis; 4, forelimbs and hind limb paralysis; and 5, dying [42]. The peak clinical score was analyzed as the highest clinical score that each mouse reached after induction. Lumbosacral spinal cords were collected at peak disease from MOG_35–55_-injected mice and on day 18 from CFA animals for analysis.

### Primary microglia culture and treatment

The preparation of mouse cortical microglia has been described previously [38]. Briefly, P1-P3 neonatal wild-type (WT) and IRAK-M^−/−^ mice were sacrificed to prepare single-cell suspensions of mixed glia from the cerebral cortex. After filtering through a 40-μm cell strainer, mixed glia were cultured in DMEM containing 10% FBS and 1% penicillin/streptomycin at 37 °C and 5% CO_2_ for 7–8 days. The cultures were shaken for 5 h at 200 rpm at 37 °C to collect purified microglia. Thereafter, both WT and IRAK-M^−/−^ microglia cultures were exposed to PBS for 5 h, or LPS (100 ng/mL) for 3.5 h and nigericin (2.5 μM) for 1 h. Finally, stimulated and unstimulated microglia were collected for western blot, RT-PCR analysis, and immunofluorescence.

### Inflammatory infiltration, LFB staining, and immunohistochemistry

The animals were sacrificed 16–20 days after immunization, and perfused using ice-cold PBS and 4% paraformaldehyde. Subsequently, their lumbosacral spinal cords were dissected and fixed in fresh 4% paraformaldehyde. Twenty-four hours later, the tissues were dehydrated with gradient alcohol and paraffin-embedded. The paraffin tissue blocks were then sectioned into 4-μm thick sections for H&E and LFB staining to evaluate inflammation and demyelination. The inflammatory infiltration evaluation was based on the previously outlined 4-or 5-point scale [43]. For immunohistochemistry, paraffin slices were deparaffinized and blocked with 10% donkey serum in TBST and subsequently incubated overnight at 4 °C with primary antibodies, including antibodies against NLRP3 (rat IgG, 1:100), ASC (rabbit IgG, 1:400), caspase-1 (rabbit IgG, 1:100), cleaved IL-1β (rabbit IgG, 1:100), GSDMD (rabbit IgG, 1:600), and Iba-1 (goat IgG, 1:400). Thereafter, the sections were washed, covered with secondary antibody, and stained with DAB. Images were then acquired by microscopy (Olympus, Tokyo, Japan).

### Immunofluorescence

The lumbosacral spinal cords were extracted and fixed as above, before dehydrating in 15% sucrose solution for 4 h, 20% sucrose solution for 10 h, and 30% sucrose solution overnight at 4 °C. Finally, the spinal cords were embedded in OCT and stored at −80 °C. For immunofluorescence assay, 5-μm thick sections from all of the groups were prepared, washed with TBST for 15 min, fixed with paraformaldehyde for 10 min, permeabilized with 0.3% Triton X-100 for 15 min, blocked with 10% normal donkey serum in TBST for 1.5 h, and incubated overnight at 4 °C with primary antibodies against NLRP3 (rat IgG, 1:150), ASC (rabbit IgG, 1:300), caspase-1 (mouse IgG, 1:80), cleaved IL-1β (rabbit IgG, 1:100), GSDMD (rabbit IgG, 1:400), and Iba-1 (goat IgG, 1:300). Following incubation, the sections were incubated with Alexa Fluor 488-, 594-, or 647-conjugated secondary antibody (1:400), before DAPI was used to stain the nuclei. Spinal cord tissues were visualized and captured with a confocal laser microscope (Nikon A1, Nikon, Tokyo, Japan) or fluorescence microscopy (Olympus, Tokyo, Japan) at the same parameters. Immunofluorescence images of cells were captured via fluorescence microscopy (Olympus, Tokyo, Japan). Immunofluorescence was analyzed by ImageJ (National Institutes of Health, Bethesda, MD) on six lumbar spinal cord sections for each littermate.

### Western blot

Primary microglia and spinal cords were lysed in RIPA buffer supplemented with 1 mM phenylmethanesulfonyl fluoride to extract proteins. The protein concentrations were quantified using a bicinchoninic acid (BCA) protein reagent kit, after which the proteins were mixed with SDS-loading buffer and subjected to thermic denaturation. Then, 40 μg of proteins of different groups were separated by SDS-PAGE on 10%–12% gels and then transferred onto polyvinylidene difluoride (PVDF) membranes. Subsequently, the membranes were blocked for 1.5 h at room temperature with 5% skim milk powder or BSA (Solarbio, China) in TBST and incubated with primary antibody (anti-NLRP3 (1:500), anti-ASC (1:500), anti-caspase-1 (1:200), anti-pro IL-1β (1:1000), anti-cleaved IL-1β (1:1000), and anti-GSDMD (1:2500) diluted in blocking solution overnight at 4 °C. Following incubation, the blots were incubated with horseradish peroxidase-conjugated secondary antibody at room temperature for another 2 h. Finally, the bands were visualized with enhanced chemiluminescence reagents.

### Real-time quantitative PCR statistical analysis

Total RNA from the lumbosacral spinal cord and primary microglia was extracted with Trizol. The total RNA (1 μg) of each sample was reverse transcribed to complementary DNA (cDNA) based on a PrimeScript RT Master Mix Kit. RT-PCR was performed using a 20 μL mixture of SYBR Premix Ex Taq II Kit, template cDNA, and appropriate primers. The primer sequences used are shown in Supplementary Table 1. The PCR condition was as follows: denaturation at 95 °C for 30 s, 40 cycles of denaturation at 95 °C for 5 s, and annealing and extension at 60 °C for 30 s. Data were determined using an ABI 7500 Real-Time PCR System (Applied Biosystems, Carlsbad, CA).

### Immunoprecipitation

Lumbosacral spinal cords collected at peak disease were lysed and pretreated with anti-IRAK1 overnight at 4 °C. Thereafter, protein A/G agarose beads were added to the lysates and incubated for 3 h, before immediately washing three times with RIPA buffer. Then, sample loading buffer was added to the samples and boiled for 10 min. Finally, similar to western blot, the samples were run on SDS-PAGE according to standard procedures.

### Intracerebroventricular injection

Three weeks before EAE modeling, 6-week-old female C57BL/6 mice were used for intracerebroventricular (ICV) injection of 1 × 10^10 ^μg vector adeno-associated virus (AAV) 9-IRAK-M, or control vector AAV9 to construct the IRAK-M overexpression group (AAV^IRAK-M^) and the corresponding control group (AAV^CTL^). ICV injections were conducted according to previous studies [38]. Briefly, mice were administered anesthesia with isoflurane, followed by fixation on stereotaxic apparatus (RWD, Shenzhen, China). Then, an incision was made to the skin on the surface of the skull to expose the bregma. The stereotaxic coordinates from the bregma were as follows: −0.21 mm anteroposterior, −0.95 mm mediolateral, and −2.38 mm dorsoventral. Three weeks later, before EAE modeling, the expression of IRAK-M mRNA was detected by RT-PCR.

### Statistical analysis

Statistical analysis was performed using GraphPad Prism 8 (GraphPad, La Jolla, CA, US) and SPSS version 24.0 (IBM, Armonk, NY, USA). Data are displayed as the mean ± SEM. The Kruskal–Wallis test, an unpaired *t*-test, or one-way analysis of variance (ANOVA) with post hoc multiple comparison tests were performed as indicated in the figure legends. *P*-values < 0.05 were considered statistically significant (^*^P < 0.05; ^**^P < 0.01; ^***^P < 0.001).

## Results

### NLRP3 inflammasomes and GSDMD-mediated pyroptosis were activated in the CNS microglia of EAE

**Fig. 1:**
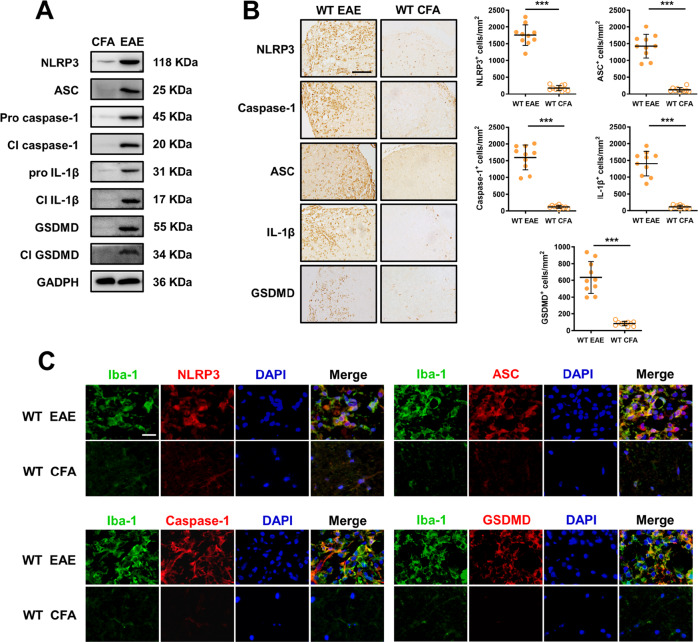
NLRP3 inflammasome and GSDMD are strongly expressed and activated in CNS microglia during EAE. **A** Immunoblot analysis of NLRP3, ASC, full-length (FL) and cleaved caspase-1, IL-1β, and GSDMD in the lumbosacral spinal cords of EAE-induced WT mice at peak disease or CFA-treated mice on day 18 after treatment (*n* = 10 per group). **B** Immunohistochemistry showing infiltration of NLRP3, ASC, caspase-1, IL-1β, and GSDMD-positive immune cells in the spinal cords of EAE-induced WT mice at peak disease or CFA-treated WT mice on day 18 after treatment. Scale bar: 50 µm. *n* = 10 per group. **C** Immunofluorescent labeling of Iba-1 (green), NLRP3, ASC, caspase-1, or GSDMD (red), and DAPI (blue) demonstrates the expression and process of NLRP3 inflammasome and GSDMD-mediated pyroptosis in the microglia of WT EAE mice at peak disease or CFA-treated WT mice on day 18 after treatment (*n* = 10 per group). Scale bar: 20 µm. Data are from three representative independent experiments and were analyzed by an unpaired *t*-test or the Mann–Whitney *U* test. Error bars show the mean ± SEM. ^***^P < 0.001.

To investigate NLRP3 inflammasome activation and GSDMD-mediated pyroptosis in EAE, we measured the levels of NLRP3 inflammasome-related molecules and GSDMD in the lumbosacral spinal cords of WT EAE and WT CFA mice using western blot and RT-PCR analysis. Our results showed that the expression of NLRP3, ASC, full-length and cleaved GSDMD, caspase-1, and IL-1β in the lumbar spinal cord of WT EAE mice was dramatically increased at the peak stage compared to that of WT mice treated with CFA (Fig. 1A), which suggested the involvement of both NLRP3 inflammasomes and GSDMD-mediated pyroptosis in EAE. To further detect the clustering of NLRP3^+^, ASC^+^, caspase-1^+^, IL-1β^+^, and GSDMD^+^ immune cells, and the degree of NLRP3 inflammasome activation and GSDMD in the microglia, we conducted immunohistochemistry on the spinal cords of WT mice administered with MOG_35–55_ or CFA. The results showed that the expression of NLRP3 inflammasomes (*P* < 0.001) and GSDMD (*P* < 0.001) in the central nervous system of EAE was greatly increased, with particularly high levels of positive immune cells, such as ASC^+^ immune cells (*P* < 0.001), caspase-1^+^ immune cells (*P* < 0.001), IL-1β^+^ immune cells (*P* < 0.001) in the lesion areas of the spinal cord, which were characterized by intense inflammation. In contrast, few positive cells were detectable in the spinal cord of CFA mice (Fig. 1B). The high expression of Iba-1 was confirmed by immunofluorescence staining in microglia of the lumbosacral spinal cord (Fig. 1C), which suggested the activation of NLRP3 inflammasomes and GSDMD-mediated pyroptosis in CNS microglia of EAE. Overall, these data revealed that NLRP3 inflammasomes and GSDMD-mediated pyroptosis are involved in the development of EAE, and that their activation in microglia may promote the deterioration of EAE.

### Deletion of IRAK-M increases the incidence, exacerbates neuroinflammation and neurological symptoms of EAE

**Fig. 2:**
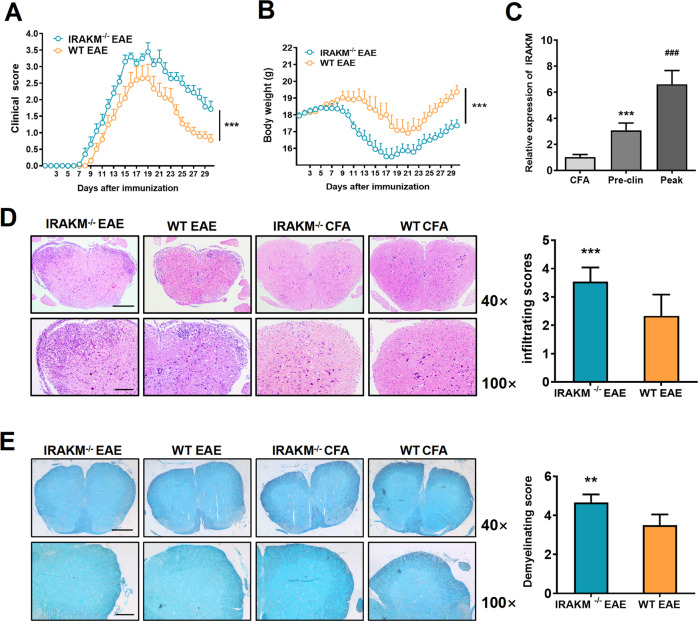
IRAK-M deficiency deteriorates neurobehavioral symptoms and pathological changes in the EAE model. **A** Average clinical scores of IRAK-M^−/−^ and WT EAE groups, respectively (*n* = 12 per group). **B** The variation of body weight of IRAK-M^−/−^ and WT EAE groups, respectively (*n* = 12 per group). **C** Relative fold induction of IRAK-M mRNA in the development of WT EAE on day 10 and at peak disease compared to WT CFA on day 18 (*n* = 5, respectively). ^***^P < 0.001 compared to the WT CFA group. ^###^*P* < 0.001 compared to WT EAE on day 10. **D** Representative images of H&E staining (upper panels on the left show a 4 × 10 magnification of the whole spinal cord, scale bar: 500 µm; lower show a 10 × 10 magnification of the anterior median region, scale bar: 200 µm). The right panel shows the evaluation of infiltrating degree (*n* = 12 per group). **E** Representative images of LFB staining. The uncolored area in the anterior white matter indicates the area of axonal injury (upper panels on the left show a 4 × 10 magnification of the whole spinal cord, scale bar: 500 µm; lower show a 10 × 10 magnification of the anterior median region, scale bar: 200 µm). The right panel shows the evaluation of the demyelination degree (*n* = 12 per group). Results were normalized to GAPDH. All results were from three representative experiments. Data show the mean ± SEM and were analyzed by an unpaired *t*-test (**A**, **B**, **D**, **E**) or *t*he Mann–Whitney *U* test (**C**). ^**^P < 0.01; ^***^P < 0.001.

The subsequent experiments were performed to determine the role of IRAK-M in regulating the severity of EAE. Compared to WT littermate mice, IRAK-M^−/−^ mice were more vulnerable to EAE, mainly presenting as increased incidence, weight loss (*P* < 0.001), severe inflammatory infiltration (*P* < 0.001), and improved clinical symptoms (*P* < 0.001; Fig. 2A, B, D). Furthermore, to determine the changes in mRNA expression of IRAK-M during EAE modeling in vivo, we used the mRNA of lumbosacral spinal cords from WT EAE mice on day 10 and at peak disease, and WT CFA mice on day 18 for RT-PCR analysis. Following the induction of EAE, the expression levels of IRAK-M in WT mice were generally elevated and reached the highest levels at peak disease (*F* (2,12) = 82.40; *P* < 0.001) (Fig. 2C). Additionally, we evaluated the extent of inflammatory infiltration by scores. H&E staining with spinal cord sections of IRAK-M^−/−^ animals at peak disease revealed signs of severe neuritis, as indicated by immune cell clustering and disorganization compared to the lower infiltration observed in WT mice (*P* < 0.001) during EAE (Fig. 2D). LFB staining was performed for demyelination access, showing severe degrees of demyelination in the spinal cords of IRAK-M^−/−^ mice (*P* < 0.001) relative to their WT counterparts during EAE (Fig. 2E). These data suggest that IRAK-M^−/−^ mice are more susceptible to EAE.

### IRAK-M modified the expression of NLRP3 inflammasomes and GSDMD in the CNS

**Fig. 3:**
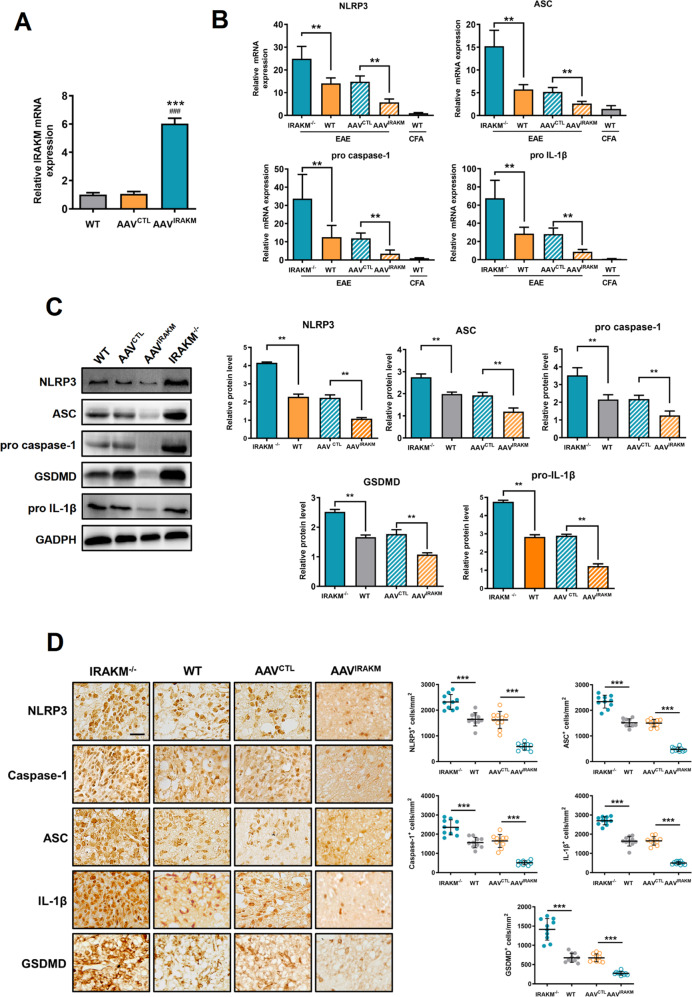
IRAK-M reduces gene and protein expressions of NLRP3 inflammasome, IL-1β, and GSDMD in the lumbosacral spinal cord during peak disease. **A** Relative fold expression of IRAK-M mRNA in the spinal cord of AAV^IRAK-M^ mice compared to AAV^CTL^ and WT mice before EAE immunization (*n* = 5, respectively). ***P < 0.001 compared to the CFA group. ^###^*P* < 0.001 compared to the AAV^CTL^ group. **B** Quantification of mRNA expression levels of NLRP3 inflammasome-related genes were assessed by RT-PCR using lumbosacral spinal cord obtained at peak disease from IRAK-M^−/−^ EAE and WT EAE groups (*n* = 6, respectively). Values represent relative fold changes compared to AAV^IRAK-M^ EAE mice and were normalized to GAPDH. Intravenously injected AAV with IRAK-M suppressed the CNS infiltration of NLRP3 inflammasome-related cells and ameliorated EAE. **C** The left panel shows representative western blotting images of indicated protein levels in lumbosacral spinal cord slices at peak disease from WT + EAE, IRAK-M^−/−^ + EAE, AAV^CTL^ + EAE, and AAV^IRAK-M^ + EAE mice. The right panel shows quantification analysis of western blot (normalized to GADPH) (*n* = 4 per group). **D** Immunohistochemistry was performed to detect the expression of NLRP3, ASC, caspase-1, GSDMD, and IL-1β in the lumbosacral spinal cord. The left panel shows the representative images. The right panel shows the quantification of cell numbers. Three sections per mouse were evaluated (*n* = 4 per group). Scale bar: 50 μm. Kruskal–Wallis plus Dunn’s test or one-way ANOVA, followed by Tukey’s post hoc analysis, was used for quantification. All data are shown as mean ± SEM. ^**^P < 0.01, ^***^P < 0.001.

To evaluate the role of IRAK-M in regulating the expression of the NLRP3 inflammasome and GSDMD in the CNS during EAE, we administered MOG_35-55_ to the littermates of IRAK-M-knockout mice, WT, AAV^CTL^, and AAV^IRAK-M^ mice. To confirm the successful establishment of AAV^IRAK-M^ mice, we detected the mRNA levels of IRAK-M using lumbosacral spinal cords taken from WT, AAV^CTL^, and AAV^IRAK-M^ mice, which showed that IRAK-M was over-expressed in the AAV^IRAK-M^ group (*F* (2, 12) = 653.9; *P* < 0.001) relative to the other two counterparts (Fig. 3A). Unsurprisingly, the expression levels of NLRP3 inflammasome and GSDMD in the CNS assessed by RT-PCR showed the highest expression of NLRP3 (*F*(4, 25) = 58.24; *P* < 0.001), ASC (*F*(4, 25) = 58.47; *P* < 0.001), caspase-1 (*F*(4, 25) = 21.71; *P* < 0.001), and IL-1β (*F*(4, 25) = 40.98; *P* < 0.001) in IRAK-M^−/−^ mice, while the lowest in AAV^IRAK-M^ mice compared to WT and AAV^CTL^ groups, and the results of immunoblotting analysis also demonstrated that NLRP3 (*F*(3, 12) = 122.8; *P* < 0.001), ASC (*F* (3, 12) = 84.82; *P* < 0.001), pro caspase-1 (*F*(3, 12) = 38.20; *P* < 0.001), pro IL-1β (*F*(3, 12) = 180.9; *P* < 0.001), and GSDMD (*F*(3, 12) = 148.0; *P* < 0.001) had a trend consistent with RT-PCR among groups, revealing the negative role of IRAK-M in modifying both gene and protein expression of the NLRP3 inflammasome and GSDMD in the CNS of EAE (Fig. 3B, C). In concordance with this result, immunohistochemical analysis further demonstrated that most immune cells infiltrated in the CNS of IRAK-M^-/-^ mice, with increased expression of NLRP3 (*F*(3, 36) = 73.67; *P* < 0.001), ASC (*F*(3, 36) = 203.0; *P* < 0.001), caspase-1 (*F*(3, 36) = 65.35; *P* < 0.001), IL-1β (*F*(3, 36) = 198.8; *P* < 0.001), and GSDMD (*F*(3, 36) = 86.34; *P* < 0.001) in the spinal cord compared to WT littermates during EAE, while this phenomenon was suppressed by overexpression of IRAK-M. Relatively few microglia were activated in AAV^IRAK-M^ mice, which is similar to that observed in CFA mice (Fig. 3D).

### IRAK-M deficiency promotes activation of microglia, NLRP3 inflammasome, and GSDMD-mediated pyroptosis in the microglia of EAE

**Fig. 4:**
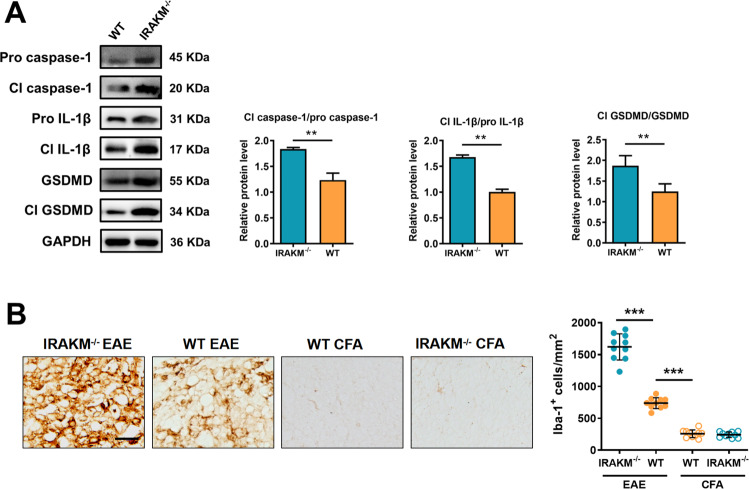
IRAK-M deficiency promotes activation of microglia, NLRP3 inflammasomes, and GSDMD-mediated pyroptosis in the microglia of EAE. **A** Western blotting of full-length (FL) and cleaved caspase-1, IL-1β, and GSDMD. The corresponding charts show the quantification of target protein expression (*n* = 4 per group). **B** Immunohistochemistry for the detection of Iba-1^+^ microglia infiltrated in the lumbosacral spinal cord at peak disease. The left panel shows representative images. Scale bar: 50 μm. The right panel shows the quantification of cell numbers, respectively. Three sections per mouse were evaluated (*n* = 4 per group). Data were analyzed by an unpaired *t*-test or the Mann–Whitney *U* test and are shown as the mean ± SEM. ^**^P < 0.01, ^***^P < 0.001.

Microglia are the resident macrophages in the CNS. As the main innate immune cells in the CNS, microglia play a key role in the inflammatory process of EAE and have aroused increasing attention. To investigate the influence of IRAK-M on microglia, we evaluated the activation status of microglia in the lumbosacral spinal cord. Immunohistochemical analysis confirmed an increase in microglia (Iba-1^+^) infiltrated in the lumbosacral spinal cord of IRAK-M^−/−^ mice (*F*(3, 36) = 303.1; *P* < 0.001) relative to the WT group after EAE immunization, especially in lesion areas of the spinal cord (Fig. 4B). Thus, it is revealed that IRAK-M deletion promotes microglia-related inflammation in an EAE model. Western blotting demonstrated greatly increased expression of cleaved GSDMD (*P* < 0.01), cleaved caspase-1 (*P* < 0.01), and cleaved IL-1β (*P* < 0.01) in the CNS of EAE-inducted IRAK-M^−/−^ mice than that of WT mice at the peak stage (Fig. 4A). The ELISA results showed an increased level of IL-1β and TNF-α in the lumbosacral spinal cord of IRAK-M^−/−^ mice compared to WT mice after EAE (Supplementary Fig. 1). These findings suggest that the deficiency of IRAK-M can promote NLRP3 inflammasome-related activity and GSDMD-mediated pyroptosis in this disease. Additionally, confocal immunofluorescence analysis of lumbosacral spinal cords further detected an elevated degree of NLRP3 inflammasome activation (*P* < 0.001) and increased GSDMD (*P* < 0.001), ASC (*P* < 0.001), and caspase-1 (*P* < 0.001) expression in activated microglia of IRAK-M^−/−^ mice compared to that of WT mice after EAE modeling (Supplementary Fig. 2).

### Over-expression of IRAK-M suppresses neuroinflammation, NLRP3 inflammasome-related activity and GSDMD-mediated pyroptosis in CNS microglia of EAE

**Fig. 5:**
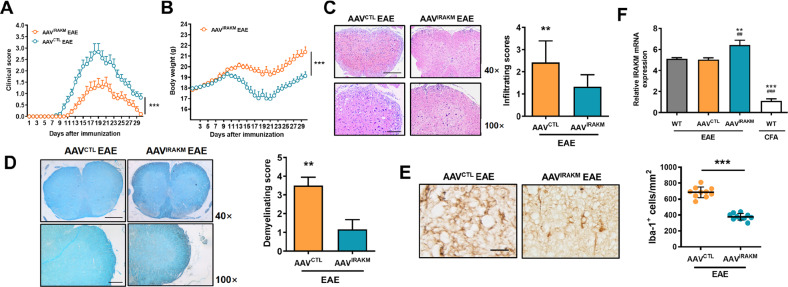
IRAK-M downregulates the expression and activation of NLRP3 inflammasomes and GSDMD. **A** Average clinical scores of AAV^IRAK-M^ and AAV^CTL^ mice administered MOG_35-55_ (*n* = 12 per group). **B** The variation in the body weight of AAV^IRAK-M^ and AAV^CTL^ EAE groups (*n* = 12 per group). **C** Representative images of H&E staining. Upper panels on the left show a 4 × 10 magnification of the whole spinal cord, scale bar: 500 µm; while the lower show a 10 × 10 magnification of the anterior median region, scale bar: 200 µm. The right panel shows the evaluation of infiltrating degree (*n* = 12 per group). **D** Representative images of LFB staining. The uncolored area in the anterior white matter indicates the area of axonal injury (upper panels on the left show a 4 × 10 magnification of the whole spinal cord, scale bar: 500 µm; lower show a 10 × 10 magnification of the anterior median region, scale bar: 200 µm). The right panel shows the evaluation of demyelination degree (*n* = 12 per group). **E** Immunohistochemistry analysis showing infiltrating Iba-1^+^ cells in the lumbosacral spinal cord of AAV^IRAK-M^ and AAV^CTL^ EAE mice. Scale bar: 50 μm. **F** Relative fold expression of IRAK-M mRNA in the spinal cord of AAV^IRAK-M^ and AAV^CTL^ mice at peak disease (the right panel) (*n* = 6, respectively). Results were normalized to GAPDH. **P < 0.01, ***P < 0.001 compared to the WT EAE group. ^##^*P* < 0.01, ^###^*P* < 0.001 compared to the AAV^CTL^ EAE group. All of the results were from three representative experiments. Data were analyzed by an unpaired *t*-test or the Mann–Whitney *U* test and are shown as the mean ± SEM. ^**^P < 0.01, ^***^P < 0.001.

To confirm the protective role of IRAK-M in EAE, WT mice were injected intracerebroventricularly with 1 × 10^10^ μg AAV 9-IRAK-M 3 weeks before EAE immunization, which is taken as the AAV^IRAK-M^ group. The control mice were injected with control virus (AAV^CTL^) without IRAK-M plasmid. The results showed that AAV^IRAK-M^ mice were more EAE-resistant, showed milder symptoms, heavier weight (*P* < 0.001), and lower clinical scores (*P* < 0.001) than AAV^CTL^ mice (Fig. 5A, B). Moreover, the expression level of IRAK-M was higher in the AAV^IRAK-M^ group than that in the AAV^CTL^ group, which is consistent with the lower clinical scores in the AAV^IRAK-M^ group (*F*(3, 20) = 440.0; *P* < 0.001) than that in the AAV^CTL^ group, suggesting that IRAK-M protects mice from disease (Fig. 5F). Additionally, IRAK-M ^−/−^ mice showed the earliest clinical symptoms and reached the disease peak quicker, and the peak duration time of the IRAK-M^−/−^ group was longer than that of other groups (Supplementary Table 2). Histological analysis showed the higher infiltration (*P* < 0.001) and demyelination (*P* < 0.001) of AAV^CTL^ mice compared to AAV^IRAK-M^ mice (Fig. 5C, D). Similarly, AAV^IRAK-M^ animals presented much less infiltration of Iba1^+^ cells in the lesion areas of the spinal cord relative to the AAV^CTL^ group, revealing that the upregulation of Iba1^+^ microglia in EAE can be reversed by overexpression of IRAK-M (Fig. 5E). Importantly, higher levels of IL-1β and TNF-α in AAV^CTL^ mice were detected compared to AAV^IRAK-M^ groups during EAE (Supplementary Fig. 1). Moreover, confocal immunofluorescence showed elevated activation of NLRP3 inflammasomes (*P* < 0.001) and GSDMD-mediated pyrotosis in microglia in the CNS of EAE-induced AAV^CTL^ mice at the peak stage compared to AAV^IRAK-M^ mice (Supplementary Fig. 3). Taken together, our data suggest that IRAK-M positively modified EAE autoimmune responses by suppressing neuroinflammation, NLRP3 inflammasome-related activity, and GSDMD-mediated pyroptosis in microglia.

### IRAK-M deficiency exacerbates NLRP3 inflammasome-related activity and GSDMD-mediated pyroptosis in primary microglia

**Fig. 6:**
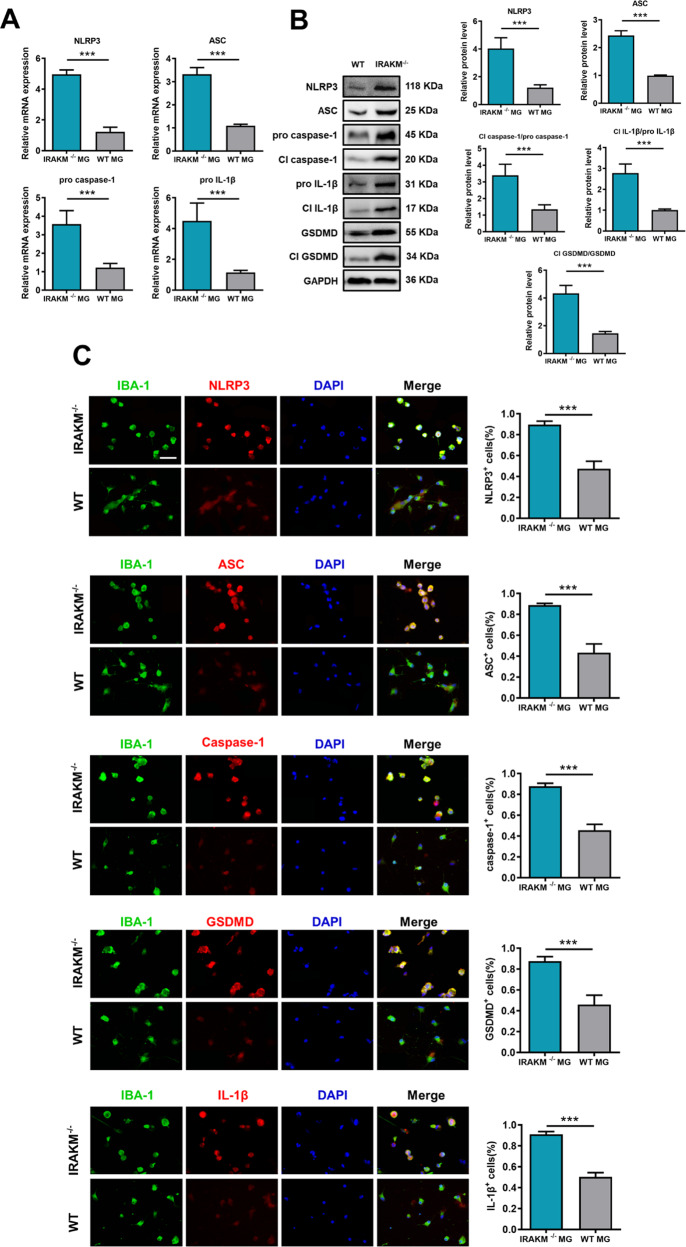
Microglia-specific IRAK-M ablation aggravates NLRP3 inflammasome-related activity and GSDMD-mediated pyroptosis in vitro. Stimulation of 100 ng/mL LPS for 3.5 h followed by 2.5 μM nigericin for 1 h was performed to mimic EAE pyroptosis in primary microglia. **A** Whole-cell lysate was collected for RT-PCR. The mRNA levels of NLRP3, ASC, cleaved caspase-1, cleaved GSDMD, and cleave IL-1β are presented as relative expression compared to WT microglia (MG) (*n* = 5 per group). **B** Western blotting of microglial protein levels of NLRP3, ASC, full-length (FL) and cleaved caspase-1, IL-1β, and GSDMD. The corresponding charts show the quantification of target protein expression (*n* = 4 per group). **C** Representative immunofluorescent analysis of NLRP3, ASC, caspase-1, IL-1β, and GSDMD in microglia. Scale bar: 20 μm. The percentage of NLRP3^+^, ASC^+^, IL-1β^+^, caspase-1^+^, and GSDMD^+^ microglia counted from ~160 microglia of five independent cultures. Unpaired *t*-tests or the Mann–Whitney *U* test were applied, and data are shown as the mean ± SEM. ^***^P < 0.001.

The dramatic activation of the NLRP3 inflammasome and GSDMD-mediated pyroptosis in IRAK-M^−/−^ mice indicates that the absence of IRAK-M might influence EAE by modifying the immune system and inflammation. We next conducted in vitro experiments with microglia taken from WT and IRAK-M^−/−^ mice to determine whether microglia are the main sites in which IRAK-M influenced NLRP3 inflammasome-related activity and GSDMD-mediated pyroptosis. As LPS plus nigericin induces pyroptosis of microglia [21], we stimulated microglia with 100 ng/mL LPS for 3.5 h before stimulation with 2.5 μM of nigericin for 1 h. Western blot, RT-PCR, and immunofluorescence analyses were performed to examine the expression of markers for NLRP3 inflammasomes and pyroptosis. RT-PCR analysis showed increased gene expression of GSDMD (*P* < 0.001), pro caspase-1 (*P* < 0.001), and pro IL-1β (*P* < 0.001) in LPS plus nigericin-treated WT microglia compared to the control (Fig. 6A), indicating the activation of NLRP3 inflammasomes and GSDMD-mediated pyroptosis in LPS plus nigericin-treated WT microglia. Additionally, a dramatic increase in protein expression of NLRP3 (*P* < 0.001), ASC (*P* < 0.001), cleaved IL-1β (*P* < 0.001), cleaved GSDMD (*P* < 0.001), and cleaved caspase-1 (*P* < 0.001) were found in IRAK-M^−/−^ microglia treated with LPS plus nigericin relative to the WT microglia (Fig. 6B), revealing that IRAK-M deletion promotes activation of both NLRP3 inflammasome and GSDMD immunoreactivity in microglia. Immunofluorescence detected obvious NLRP3 inflammasome (*P* < 0.001) and GSDMD (*P* < 0.001) immunoreactivity in the microglia of IRAK-M deficient mice compared to that in WT mice after treatment with LPS plus nigericin (Fig. 6C). Together, LPS plus nigericin exposure induces inflammasome activation and pyroptosis in microglia, which is aggravated by IRAK-M aberration. This finding suggests that IRAK-M protects microglia from NLRP3 inflammasome-related activity and GSDMD-mediated pyroptosis, and that this function of IRAK-M is responsible for inhibiting the neuroinflammation and pathogenesis of EAE.

### IRAK-M processes NLRP3 inflammasome-related activity and GSDMD-mediated pyroptosis by preventing IRAK1 phosphorylation and IRAK1/TRAF6 combination

**Fig. 7:**
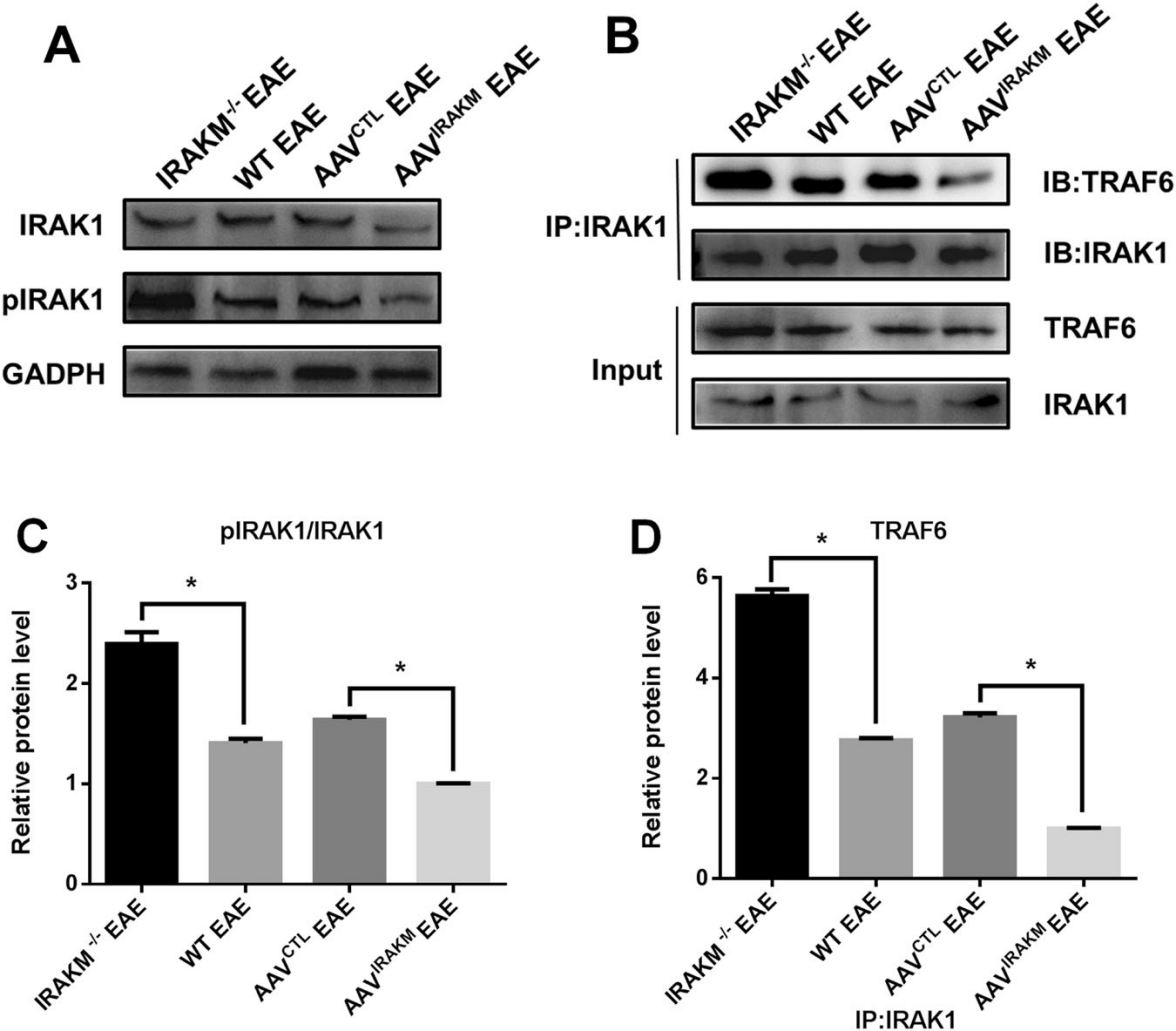
IRAK1/TRAF6 combination triggers NLRP3 inflammasome-dependent activities and GSDMD-mediated pyroptosis in EAE. **A** Western blot was performed to detect the expression of pIRAK1 and IRAK1 in the lumbosacral spinal cord from all EAE groups at peak disease. **B** Co-immunoprecipitation was performed by precipitating with anti-IRAK1, followed by western blotting for IRAK1 and TRAF6. One-way ANOVA followed by Tukey’s post hoc analysis was used for RT-PCR and western blot quantification analyses. **C** The quantification of protein expression pIRAK1/IRAK1(*n* = 5 per group). **D** The immunoprecipitation quantification of IRAK1/TRAF6 combination (*n* = 5 per group). Data are shown as the mean ± SEM. ^*^P < 0.05.

To further determine the inhibitory effect of IRAK-M on the NLRP3 inflammasome and GSDMD-mediated pyroptosis in CNS microglia of EAE, we explored the binding of IRAK1 and TRAF6, which may be the link between IRAK-M and NLRP3-related activity. IRAK-M acts as the main inhibitor of the TLR4/IL-1R signaling pathway through suppression of IRAK1 phosphorylation and its association with TRAF6 [44–46]. We hypothesize that IRAK-M protects mice from EAE by inhibiting the activation of NLRP3 inflammasomes induced by the IRAK1/TRAF6 complex. We proposed to verify whether the inhibition of the IRAK1/TRAF6 complex attributed to the effect of IRAK-M on stimulation of NLRP3 inflammasome activation and GSDMD-mediated pyroptosis. Interestingly, the levels of IRAK1 were not significantly different among different EAE groups; however, the levels of pIRAK1 were significantly increased in IRAK-M^−/−^ + EAE mice and lowest in AAV^IRAK-M^ + EAE, with WT + EAE and AAV^CTL^ + EAE showing moderate expression (Fig. 7A, C). As IRAK1 phosphorylation is an imperative factor for IRAK1 to be released from MyD88 and subsequently combines with TRAF6, immunoprecipitation analyses were performed to elucidate the connection between IRAK1 and TRAF6. Importantly, the result depicted upregulated binding of IRAK1 and TRAF6 in IRAK-M^−/−^ EAE mice compared to WT mice, which was blocked in AAV^IRAK-M^ EAE mice with enforced expression of IRAK-M post ICV injection with AAV containing IRAK-M (Fig. 7B, D). This is consistent with the elevated NLRP3 inflammasome-related activity and GSDMD-mdediated pyroptosis in IRAK-M^−/−^ EAE mice compared to other littermates. Taken together, our results suggest that IRAK-M suppressed the activation of NLRP3 inflammasomes and GSDMD-mediated pyroptosis via the inhibition of IRAK1 phosphorylation and IRAK1/TRAF6 combination during EAE.

## Discussion

**Fig. 8:**
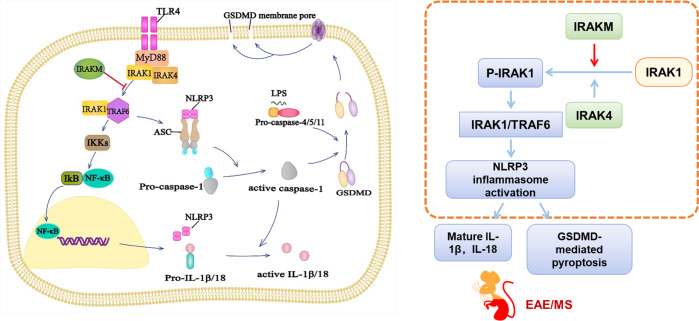
Schematic and flow diagrams illustrate the inhibited function of IRAK-M on activation of NLRP3 inflammasomes and GSDMD-mediated pyroptosis via blocking IRAK1/TRAF6 formation. TLR4 signaling is activated post EAE induction, followed by the formation of the MyD88/IRAK4/IRAK1 complex. Immediately after phosphorylation of IRAK1, IRAK1 leaves the complex and associates with TRAF6, which ultimately activates the NF-κB signaling pathway. The stimulus from TLR4 signaling rapidly promotes NLRP3, ASC, and pro-caspase-1 accumulation to form a cytosolic oligomer, which allows the self-cleavage of pro-caspase-1. Cleaved caspase-1 together with caspase 4/5/11 cleave gasdermin D (GSDMD) to generate the N-terminal domain of GSDMD, which acts as a pyroptosis executor and facilitates the release the proinflammatory cytokines IL-1β and IL-18. Inflammation and pyroptosis cause injury to microglia in the CNS of EAE. IRAK-M works as a negative regulator of the TLR4-NLRP3 inflammation pathway via inhibition of IRAK1 phosphorylation and the subsequent combination of IRAK1/TRAF6.

In this study, we found that IRAK-M deletion exacerbates the clinical symptoms of EAE and elucidated a novel role of microglial IRAK-M in resistance to IRAK1/TRAF6 complex-dependent NLRP3 inflammasome activation and pyroptosis in EAE, suggesting the potential therapeutic target for IRAK-M. Summary of the current findings is shown in Fig. 8.

MS is a multifocal demyelination disease characterized by severe inflammation, whose inflammatory extent in the CNS positively affects the degree of demyelination and disease severity [1, 2]. It is widely accepted that the TLR4 signaling pathway is involved in EAE pathogenesis by triggering inflammatory downstream signaling cascades [5, 6, 35]. TLR4 responds to stimulus post EAE induction, leading to the recruitment of IRAK1 and IRAK4 to MyD88, followed by IRAK1 and IRAK4 phosphorylation. IRAK1 then dissociates from the IRAK1/IRAK4/MyD88 myddosomes and connects to TRAF6, stimulates NF-ĸB, JNK, and MAPK pathways and release cytokines [37]. IRAK-M, as a negative regulator, disrupts IRAK1 phosphorylation, and thus prevents the dissociation of IRAK1/IRAK4/MyD88 [40, 41]. IRAK-M functions as a key signal molecule in suppressing inflammation. In response to stimulis, such as LPS and ischemia, endogenous IRAK-M is upregulated as a feedback mechanism or a compensative auto-regulation mechanism, thereby reducing inflammation [41, 47]. Consistent with these findings, our results demonstrate that the expression of IRAK-M is increased during the induction of EAE, which is consistent with the variance of IRAK-M under the stimuli of LPS and ischemia. Notably, inflammatory responses and the progression of EAE were reduced in IRAK-M^−/−^ mice compared to WT mice, while overexpression of IRAK-M inhibited the progression of EAE. This suggests that IRAK-M might protect against EAE by suppressing inflammation. Then, we performed immunoprecipitation with IRAK1 antibody to access the association between IRAK1 and TRAF6. Of note, MOG_35–55_-induced IRAK-M^−/−^ mice developed explosive activation of NLRP3 inflammasome, which is consistent with the increase in IRAK1/TRAF6 combination, indicating that IRAK1/TRAF6-mediated activation of NLRP3 inflammasomes can be blocked by IRAK-M via blocking IRAK1 phosphorylation. Here, we emphasize the protective role of IRAK-M in reducing the inflammatory response and extenuating clinical manifestations in EAE, and thus, this study provides a more comprehensive understanding of IRAK-M in inhibiting IRAK1/TRAF6-mediated signaling in EAE.

The NLRP3 inflammasome, regarded as a wide-ranging amplifier of inflammation, has been involved in diverse autoimmune states [16, 17, 24]. Multiple articles have demonstrated the critical role of the NLRP3 inflammasome in MS/EAE [48, 49]. A recent clinical study concerning variants in NLRP3 inflammasomes revealed that the activation of NLRP3 inflammasomes could result in widespread release of proinflammatory cytokines and represent a risk factor for clinical symptoms in MS [20]. Additionally, treatment targeting the NLRP3 inflammasome alleviates central neuropathic pain induced by MS [50]. Meanwhile, studies on the EAE model indicated that NLRP3 inflammasome aggregated disease via the promotion of chemotactic immune cells migrated to the CNS [29] and mediation of Th1 and Th17 responses [49, 51]. The NLRP3 inflammasome in microglia acts as a widely reactive inflammatory inducer in MS/EAE [18, 30]. Additionally, NLRP3 inflammasome-mediated pyroptosis appears in microglia in EAE mice and contributes to disease [21]. These studies underlined the importance of the NLRP3 inflammasome in the development of EAE/MS and suggested this novel complex as a possible therapeutic target for the treatment of MS. Our findings were consistent with those of previous reports, which confirmed that NLRP3 inflammasomes are elevated in EAE. Our previous study demonstrated that IRAK-M regulates disease severity by promoting M2 microglia polarization and inhibition of M1 [38]. Thus, IRAK-M may influence NLRP3 inflammasome-mediated inflammation and pyroptosis. Our results revealed that the explosive activation of NLRP3 inflammasomes in EAE mice contributed to disease severity. Importantly, this phenomenon and inflammasome-mediated pyroptosis can be prevented by enforced expression of IRAK-M, indicating that IRAK-M may protect EAE mice from suffering via promoting inactivity of NLRP3 inflammasome-related inflammation and pyroptosis.

This study highlights the pathogenic role of NLRP3 inflammasomes and pyroptosis, and the protective role of IRAK-M in EAE. Activation of the IRAK1/TRAF6 complex via TLR4 signaling could trigger NLRP3 inflammasomes to an active state through the IRAK1/NLRP3 pathway. Importantly, we revealed previously unrecognized mechanisms underlying the inhibitory function of IRAK-M in suppressing microglial NLRP3 inflammasome activation and pyroptosis by preventing the association between IRAK1 and TRAF6. Despite the beneficial function of microglial IRAK-M in EAE, the exact mechanism underlying how the IRAK1/TRAF6 complex rapidly activates remains indecipherable. To address this problem, future studies should focus on the possible intermediate process of IRAK1 activating NLRP3.

## Supplementary information

response for authorships changes

supplement data

checklist

Original Data File

## Data Availability

The datasets used or analyzed in our study are available from the corresponding author on reasonable request.

## References

[1] Dendrou CA, Fugger L, Friese MA (2015). Immunopathology of multiple sclerosis. Nat Rev Immunol.

[2] Nylander A, Hafler DA (2012). Multiple sclerosis. J Clin Invest.

[3] Cao Y, Goods BA, Raddassi K, Nepom GT, Kwok WW, Love JC (2015). Functional inflammatory profiles distinguish myelin-reactive T cells from patients with multiple sclerosis. Sci Transl Med.

[4] Park C, Ponath G, Levine-Ritterman M, Bull E, Swanson EC, De Jager PL (2019). The landscape of myeloid and astrocyte phenotypes in acute multiple sclerosis lesions. Acta Neuropathol Commun.

[5] Marta M, Meier UC, Lobell A (2009). Regulation of autoimmune encephalomyelitis by toll-like receptors. Autoimmun Rev.

[6] Marta M (2009). Toll-like receptors in multiple sclerosis mouse experimental models. Ann N Y Acad Sci.

[7] Marta M, Andersson A, Isaksson M, Kämpe O, Lobell A (2008). Unexpected regulatory roles of TLR4 and TLR9 in experimental autoimmune encephalomyelitis. Eur J Immunol.

[8] Bogie JF, Stinissen P, Hendriks JJ (2014). Macrophage subsets and microglia in multiple sclerosis. Acta Neuropathol.

[9] O’Loughlin E, Madore C, Lassmann H, Butovsky O (2018). Microglial phenotypes and functions in multiple sclerosis. Cold Spring Harb Perspect Med.

[10] Cao L, He C (2013). Polarization of macrophages and microglia in inflammatory demyelination. Neurosci Bull.

[11] Wang N, Liang H, Zen K (2014). Molecular mechanisms that influence the macrophage m1-m2 polarization balance. Front Immunol.

[12] Benedek G, Zhang J, Nguyen H, Kent G, Seifert H, Vandenbark AA (2017). Novel feedback loop between M2 macrophages/microglia and regulatory B cells in estrogen-protected EAE mice. J Neuroimmunol.

[13] Zhang J, Lapato A, Bodhankar S, Vandenbark AA, Offner H (2015). Treatment with IL-10 producing B cells in combination with E2 ameliorates EAE severity and decreases CNS inflammation in B cell-deficient mice. Metab Brain Dis.

[14] Huitinga I, van Rooijen N, de Groot CJ, Uitdehaag BM, Dijkstra CD (1990). Suppression of experimental allergic encephalomyelitis in Lewis rats after elimination of macrophages. J Exp Med.

[15] Heppner FL, Greter M, Marino D, Falsig J, Raivich G, Hövelmeyer N (2005). Experimental autoimmune encephalomyelitis repressed by microglial paralysis. Nat Med.

[16] Walsh JG, Muruve DA, Power C (2014). Inflammasomes in the CNS. Nat Rev Neurosci.

[17] Mamik MK, Power C (2017). Inflammasomes in neurological diseases: emerging pathogenic and therapeutic concepts. Brain.

[18] Liu SB, Mi WL, Wang YQ (2013). Research progress on the NLRP3 inflammasome and its role in the central nervous system. Neurosci Bull.

[19] Halle A, Hornung V, Petzold GC, Stewart CR, Monks BG, Reinheckel T (2008). The NALP3 inflammasome is involved in the innate immune response to amyloid-beta. Nat Immunol.

[20] Malhotra S, Rio J, Urcelay E, Nurtdinov R, Bustamante MF, Fernández O (2015). NLRP3 inflammasome is associated with the response to IFN-beta in patients with multiple sclerosis. Brain.

[21] McKenzie BA, Mamik MK, Saito LB, Boghozian R, Monaco MC, Major EO (2018). Caspase-1 inhibition prevents glial inflammasome activation and pyroptosis in models of multiple sclerosis. Proc Natl Acad Sci USA.

[22] Bergsbaken T, Fink SL, Cookson BT (2009). Pyroptosis: host cell death and inflammation. Nat Rev Microbiol.

[23] Ting JP, Lovering RC, Alnemri ES, Bertin J, Boss JM, Davis BK (2008). The NLR gene family: a standard nomenclature. Immunity.

[24] Davis BK, Wen H, Ting JP (2011). The inflammasome NLRs in immunity, inflammation, and associated diseases. Annu Rev Immunol.

[25] Pedra JH, Cassel SL, Sutterwala FS (2009). Sensing pathogens and danger signals by the inflammasome. Curr Opin Immunol.

[26] Jin C, Flavell RA (2010). Molecular mechanism of NLRP3 inflammasome activation. J Clin Immunol.

[27] Latz E, Xiao TS, Stutz A (2013). Activation and regulation of the inflammasomes. Nat Rev Immunol.

[28] Lawrence T (2009). The nuclear factor NF-kappaB pathway in inflammation. Cold Spring Harb Perspect Biol.

[29] Inoue M, Williams KL, Gunn MD, Shinohara ML (2012). NLRP3 inflammasome induces chemotactic immune cell migration to the CNS in experimental autoimmune encephalomyelitis. Proc Natl Acad Sci USA.

[30] Barclay W, Shinohara ML (2017). Inflammasome activation in multiple sclerosis and experimental autoimmune encephalomyelitis (EAE). Brain Pathol.

[31] Chen M, Wang H, Chen W, Meng G (2011). Regulation of adaptive immunity by the NLRP3 inflammasome. Int Immunopharmacol.

[32] Shi J, Zhao Y, Wang K, Shi X, Wang Y, Huang H (2015). Cleavage of GSDMD by inflammatory caspases determines pyroptotic cell death. Nature.

[33] Kayagaki N, Stowe IB, Lee BL, O’Rourke K, Anderson K, Warming S (2015). Caspase-11 cleaves gasdermin D for non-canonical inflammasome signalling. Nature.

[34] Kovacs SB, Miao EA (2017). Gasdermins: effectors of pyroptosis. Trends Cell Biol.

[35] Zhang Y, Han J, Wu M, Xu L, Wang Y, Yuan W (2019). Toll-like receptor 4 promotes Th17 lymphocyte infiltration via CCL25/CCR9 in pathogenesis of experimental autoimmune encephalomyelitis. J Neuroimmune Pharmacol.

[36] Akira S, Uematsu S, Takeuchi O (2006). Pathogen recognition and innate immunity. Cell.

[37] Iwasaki A, Medzhitov R (2004). Toll-like receptor control of the adaptive immune responses. Nat Immunol.

[38] Liu B, Gu Y, Pei S, Peng Y, Chen J, Pham LV (2019). Interleukin-1 receptor associated kinase (IRAK)-M -mediated type 2 microglia polarization ameliorates the severity of experimental autoimmune encephalomyelitis (EAE). J Autoimmun.

[39] Janssens S, Beyaert R (2003). Functional diversity and regulation of different interleukin-1 receptor-associated kinase (IRAK) family members. Mol Cell.

[40] Du J, Nicolaes GA, Kruijswijk D, Versloot M, van der Poll T, van ‘t Veer C (2014). The structure function of the death domain of human IRAK-M. Cell Commun Signal.

[41] Kobayashi K, Hernandez LD, Galán JE, Janeway CA, Medzhitov R, Flavell RA (2002). IRAK-M is a negative regulator of Toll-like receptor signaling. Cell.

[42] Psachoulia K, Chamberlain KA, Heo D, Davis SE, Paskus JD, Nanescu SE (2016). IL4I1 augments CNS remyelination and axonal protection by modulating T cell driven inflammation. Brain.

[43] Quan MY, Song XJ, Liu HJ, Deng XH, Hou HQ, Chen LP (2019). Amlexanox attenuates experimental autoimmune encephalomyelitis by inhibiting dendritic cell maturation and reprogramming effector and regulatory T cell responses. J Neuroinflammation.

[44] Lin KM, Hu W, Troutman TD, Jennings M, Brewer T, Li X (2014). IRAK-1 bypasses priming and directly links TLRs to rapid NLRP3 inflammasome activation. Proc Natl Acad Sci USA.

[45] Xing Y, Yao X, Li H, Xue G, Guo Q, Yang G (2017). Cutting edge: TRAF6 mediates TLR/IL-1R signaling-induced nontranscriptional priming of the NLRP3 inflammasome. J Immunol.

[46] Fernandes-Alnemri T, Kang S, Anderson C, Sagara J, Fitzgerald KA, Alnemri ES (2013). Cutting edge: TLR signaling licenses IRAK1 for rapid activation of the NLRP3 inflammasome. J Immunol.

[47] Chen W, Saxena A, Li N, Sun J, Gupta A, Lee DW (2012). Endogenous IRAK-M attenuates postinfarction remodeling through effects on macrophages and fibroblasts. Arterioscler Thromb Vasc Biol.

[48] Gharagozloo M, Gris KV, Mahvelati T, Amrani A, Lukens JR, Gris D (2017). NLR-dependent regulation of inflammation in multiple sclerosis. Front Immunol.

[49] Gris D, Ye Z, Iocca HA, Wen H, Craven RR, Gris P (2010). NLRP3 plays a critical role in the development of experimental autoimmune encephalomyelitis by mediating Th1 and Th17 responses. J Immunol.

[50] Khan N, Kuo A, Brockman DA, Cooper MA, Smith MT (2018). Pharmacological inhibition of the NLRP3 inflammasome as a potential target for multiple sclerosis induced central neuropathic pain. Inflammopharmacology.

[51] Inoue M, Shinohara ML (2013). NLRP3 Inflammasome and MS/EAE. Autoimmune Dis.

