# Supplementary figures and images for: IRAK-M suppresses the activation of microglial NLRP3 inflammasome and GSDMD-mediated pyroptosis through inhibiting IRAK1 phosphorylation during experimental autoimmune encephalomyelitis
Source: Cell Death Dis. 2023 Feb 10;14(2):103. doi: 10.1038/s41419-023-05621-6 (PMC9918485; doi:10.1038/s41419-023-05621-6)

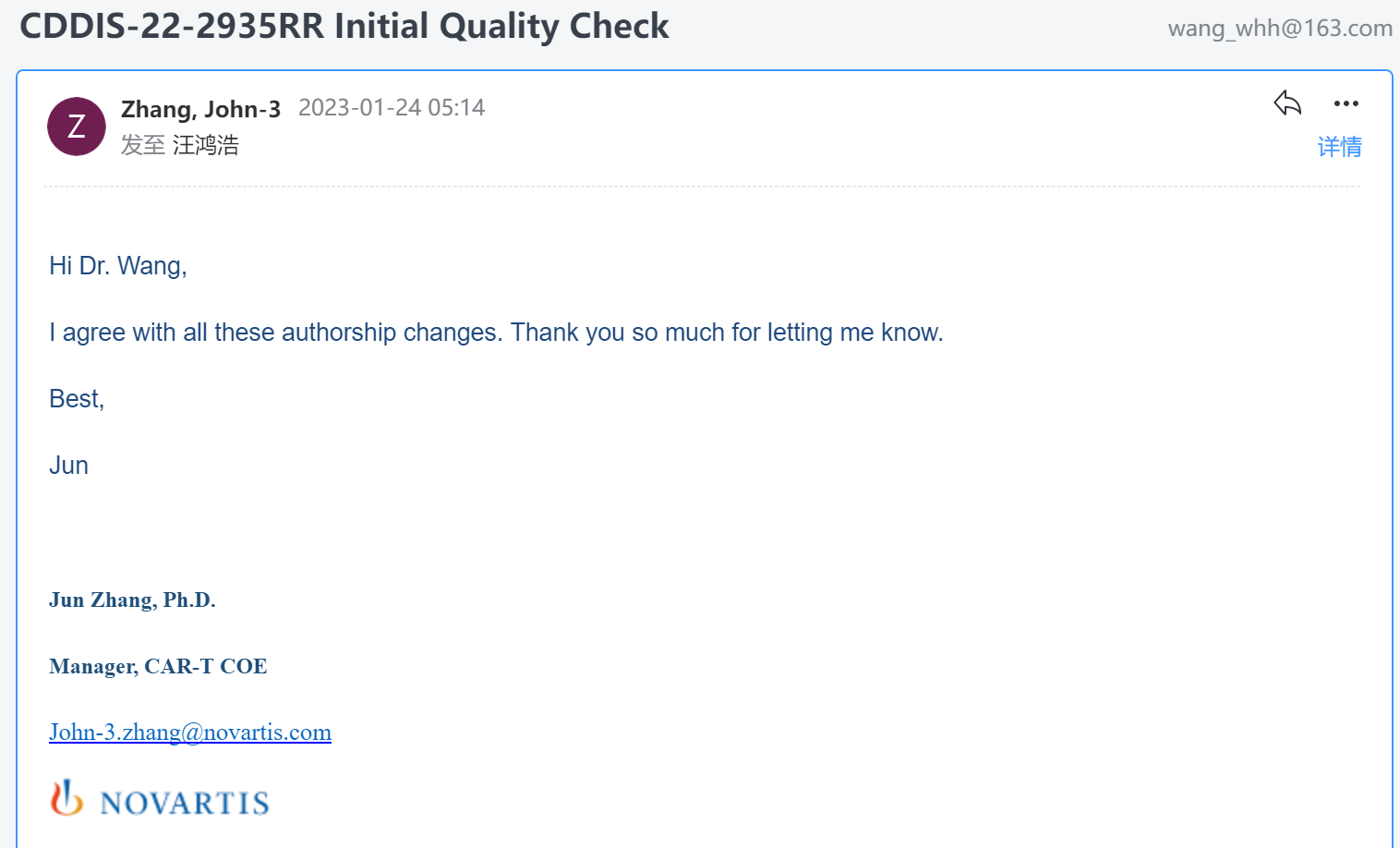


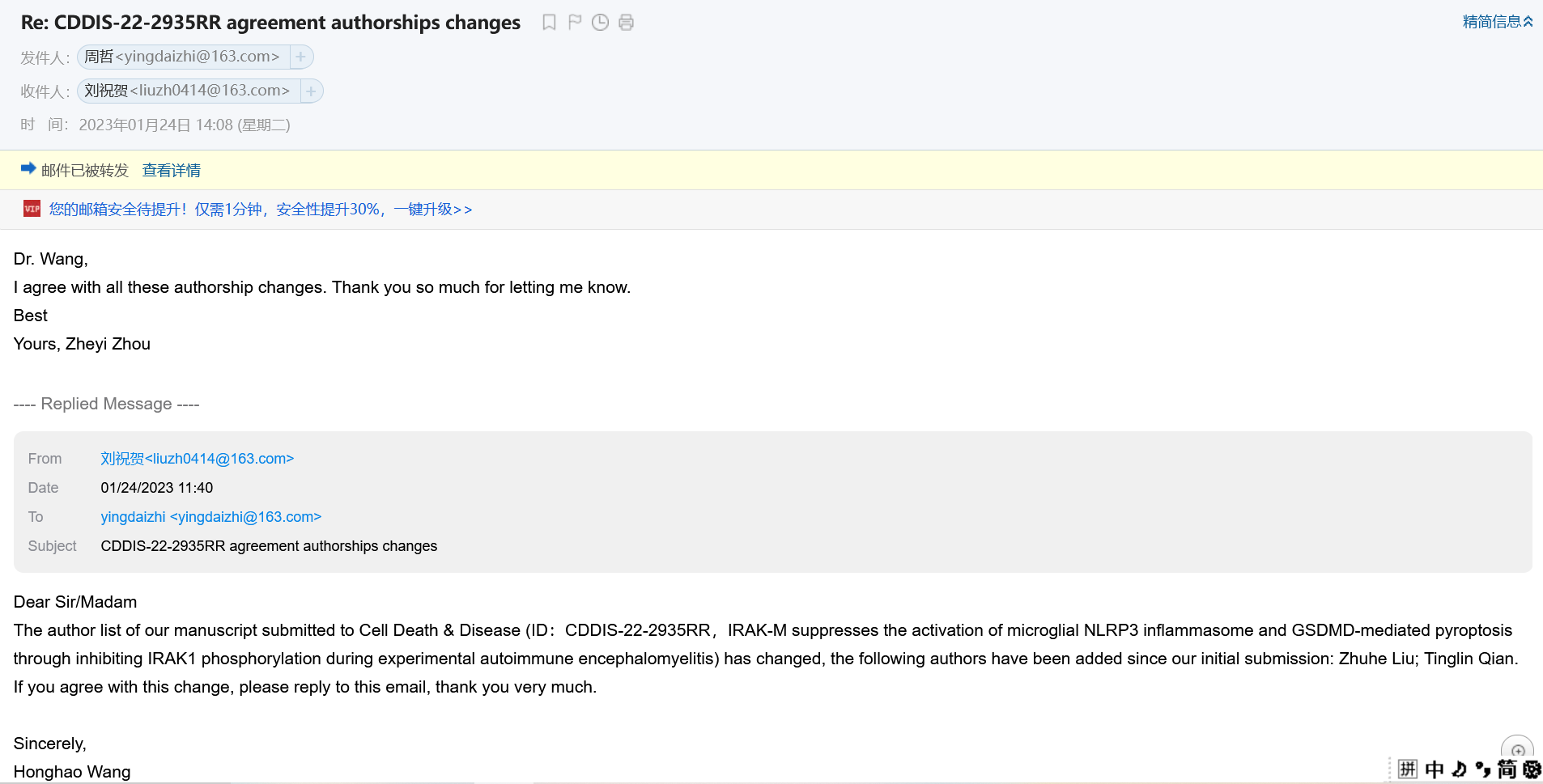

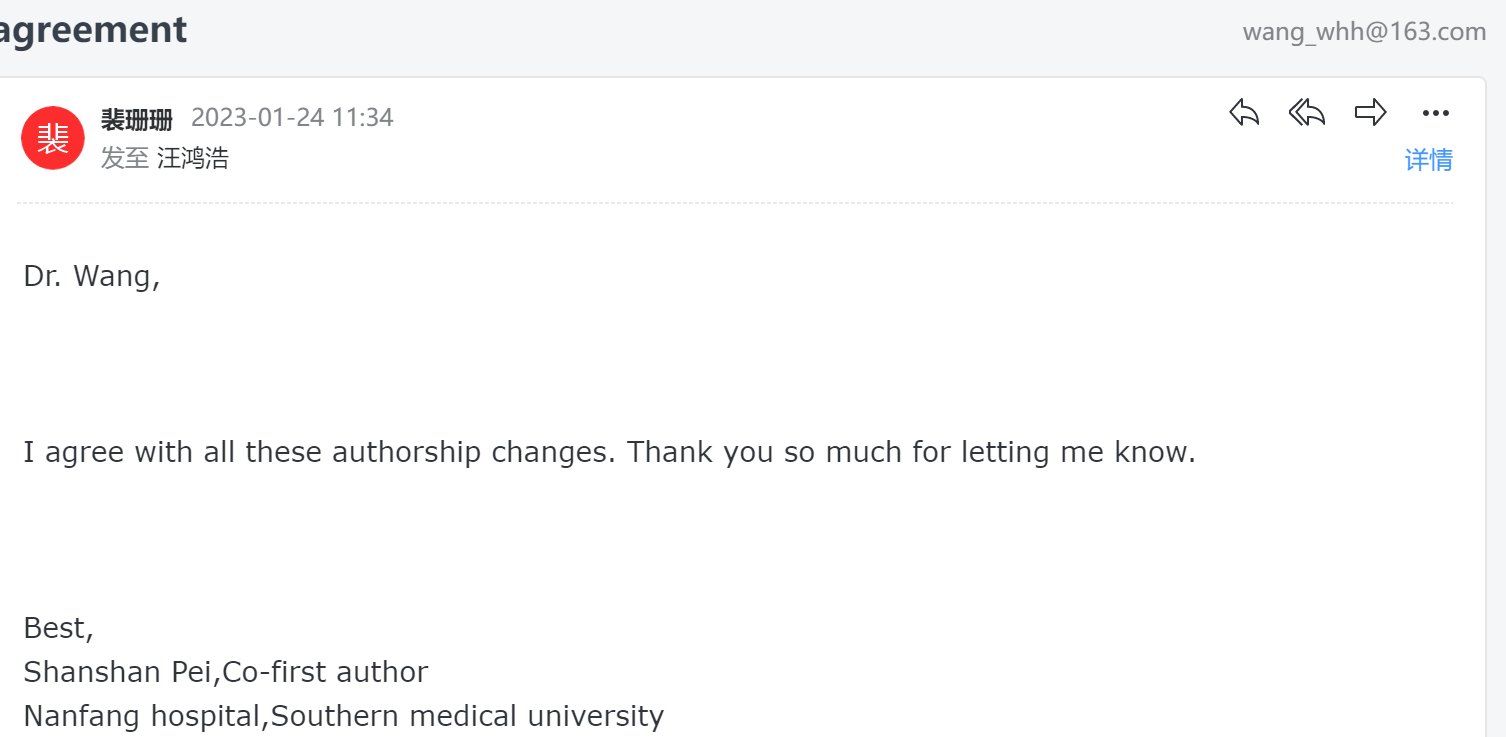


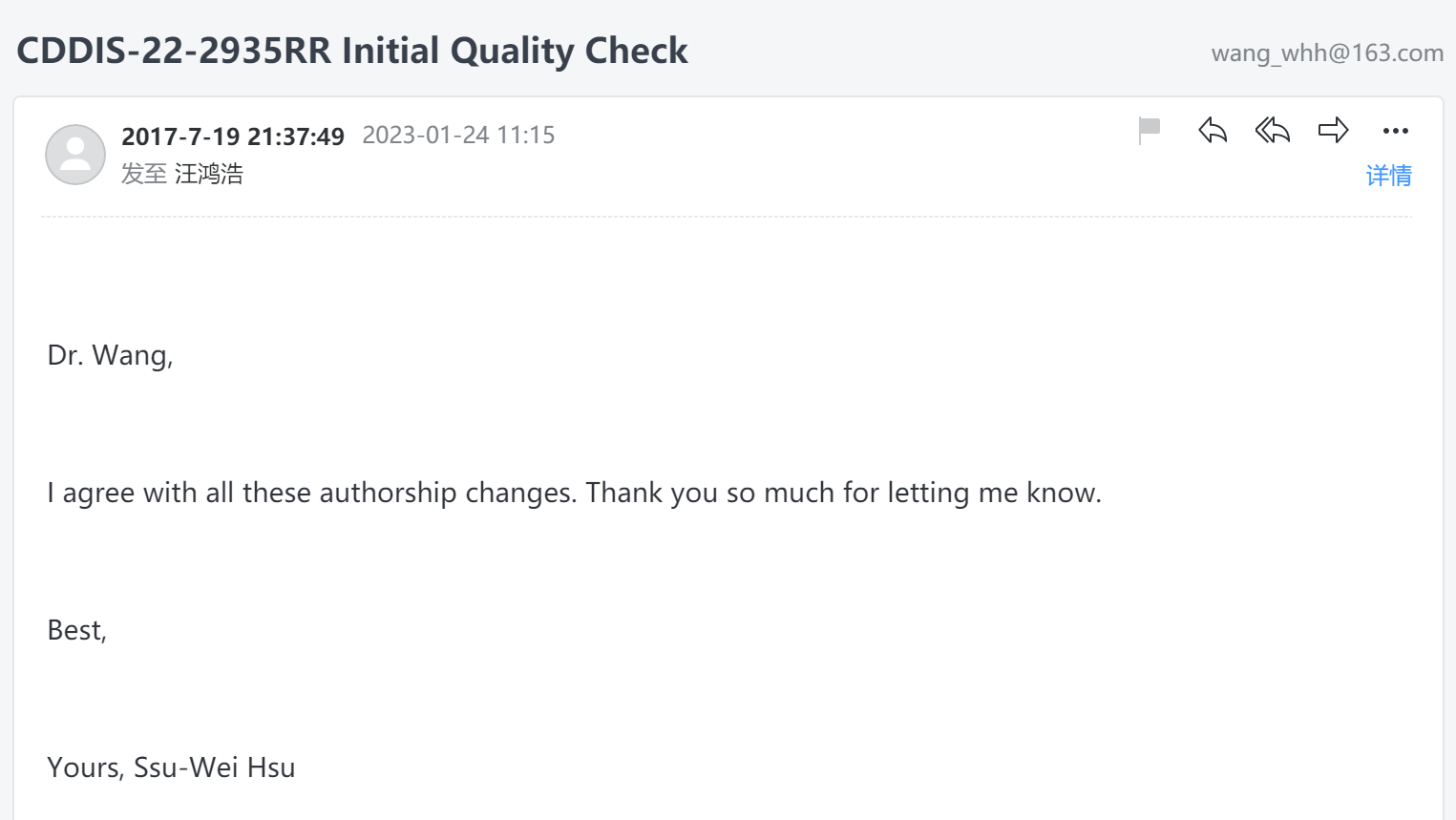


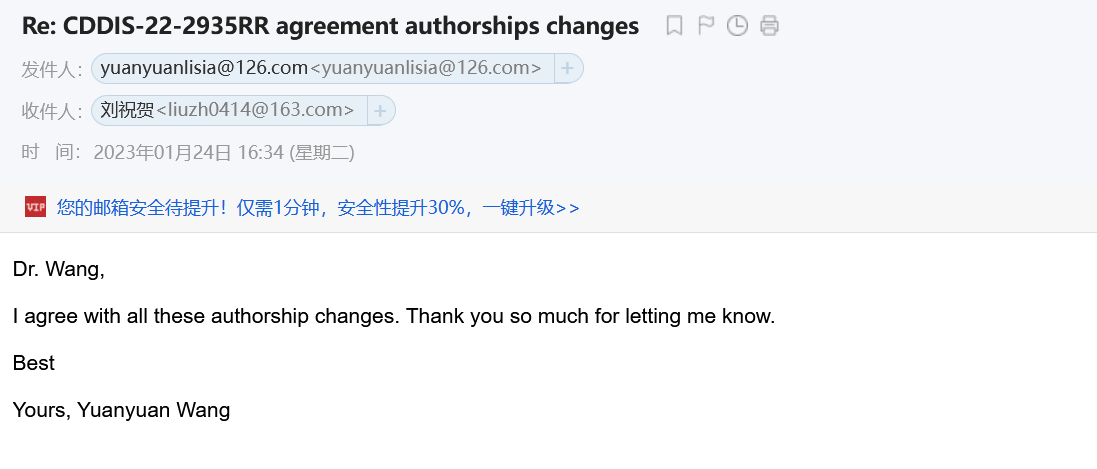


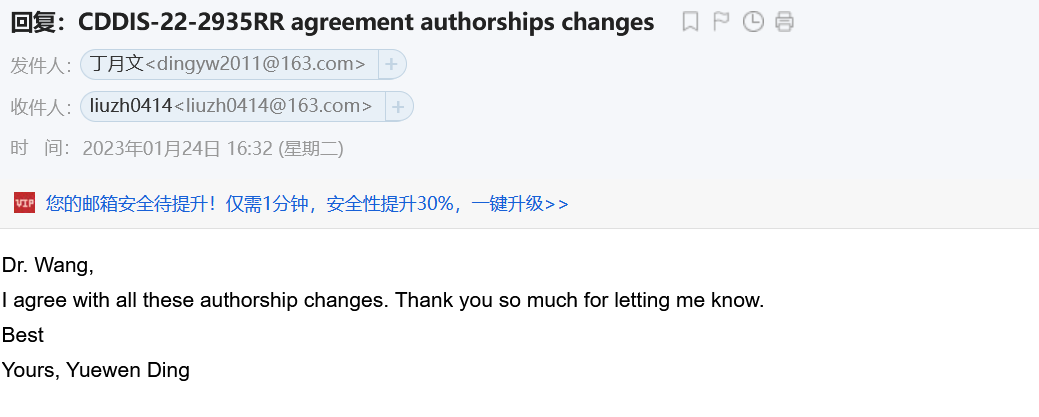


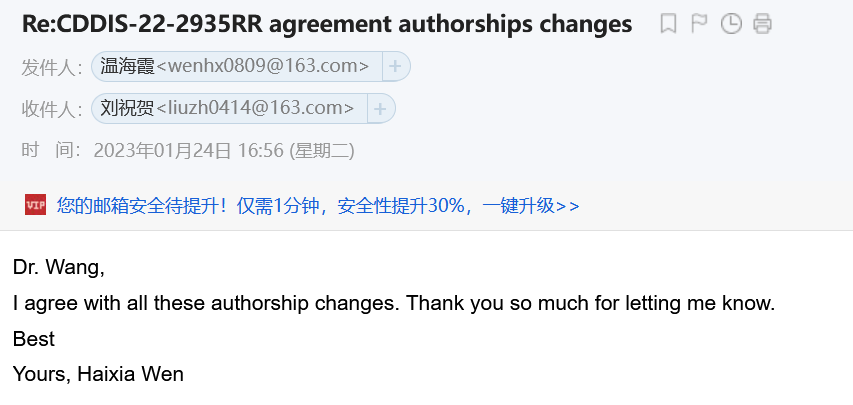

Supplement: Supplementary file 1 — response for authorships changes [file 41419_2023_5621_MOESM1_ESM.docx]
