# Supplementary material for: IRAK-M suppresses the activation of microglial NLRP3 inflammasome and GSDMD-mediated pyroptosis through inhibiting IRAK1 phosphorylation during experimental autoimmune encephalomyelitis
Source: Cell Death Dis. 2023 Feb 10;14(2):103. doi: 10.1038/s41419-023-05621-6 (PMC9918485; doi:10.1038/s41419-023-05621-6)
Supplement: Supplementary file 2 — supplement data [file 41419_2023_5621_MOESM2_ESM.docx]

Supplementary Table 1: The mouse primer sequences for Real Time PCR.

| Gene | Forward | Reverse |
| --- | --- | --- |
| NLRP3 | 5’-ATTACCCGCCCGAGAAAGG-3’ | 5’-TCGCAGCAAAGATCCACACAG-3’ |
| ASC | 5’-CTTGTCAGGGGATGAACTCAAAA-3’ | 5’-GCCATACGACTCCAGATAGTAGC-3’ |
| Caspase-1 | 5’-CCAGAGCACAAGACTTCTGAC-3’ | 5’-TGGTGTTGAAGAGCAGAAAGC-3’ |
| IL-1β | 5’-GCAACTGTTCCTGAACTCAACT -3’ | 5’-ATCTTTTGGGGTCCGTCAACT-3’ |
| IRAK-M | 5’-CAGGTGTCCTTCTCCACTGTTCTTG-3’ | 5’-GTGACCTCAGACTGGCTGCA-3’ |
| GAPDH | 5’-CAACTACATGGTCTACATGTTC-3’ | 5’-CTCGCTCCTGGAAGATG-3’ |

Supplementary Table 2: The incidence rates, survival percentage, time of disease onset and disease peak of EAE mice (n = 12 per group).

| **Group** | **Incidence (%)** | **Survival (%)** | **Day of onset (d)** | **Peak clinical score (d)** |
| --- | --- | --- | --- | --- |
| **IRAKM^-/-^ EAE** | 100 | 75 | 8.4 | 3.7 |
| **WT EAE** | 83.3 | 83.3 | 11.8 | 2.9 |
| **AAV^CTL^ EAE** | 83.3 | 83.3 | 10.5 | 3.1 |
| **AAV^IRAKM^ EAE** | 41.7 | 100 | 13 | 2.2 |

Supplementary Figure 1


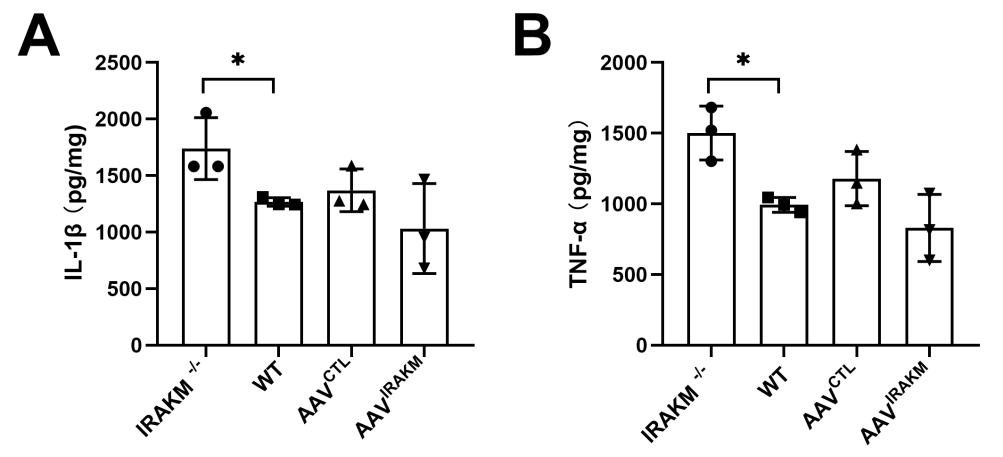


**Figure S1** The levels of IL-1β and TNF-α in the lumbosacral spinal cord of IRAK-M^-/-^ mice, WT mice, AAV^CTL^ mice, and AAV^IRAKM^ mice after EAE measured by ELISA (n = 3 per group). Data are shown as the mean ± SEM, ^*^*P* < 0.05, versus the indicated group.

Supplementary Figure 2


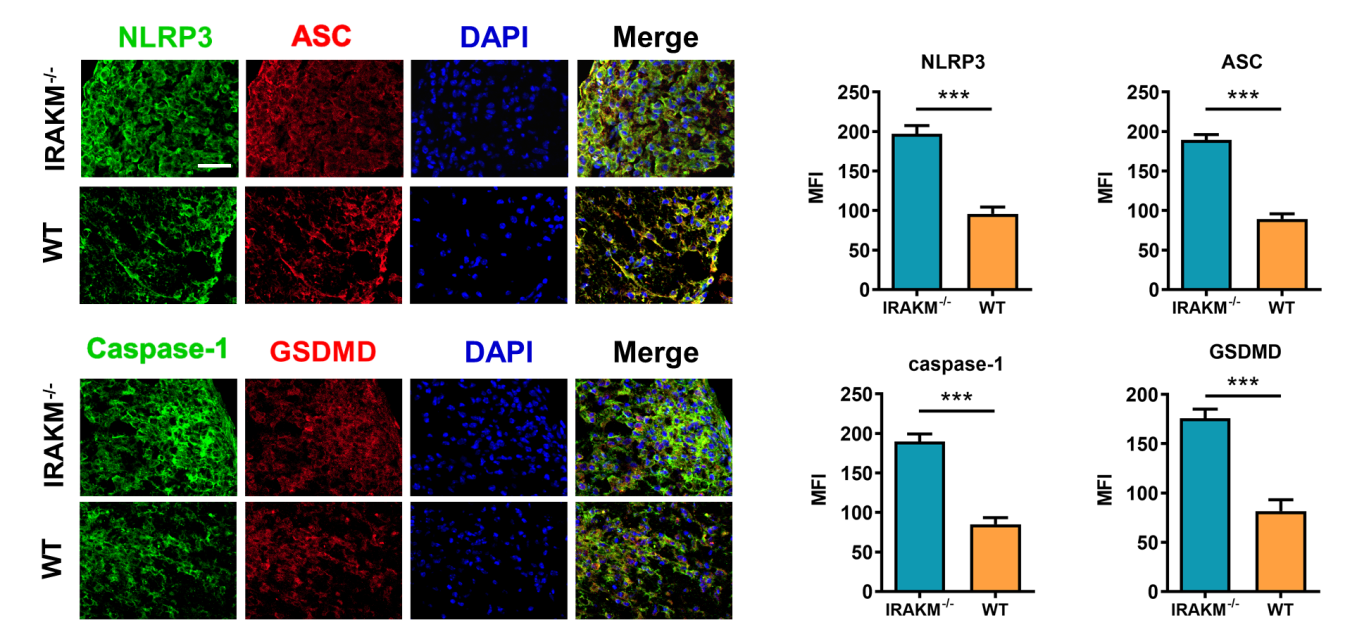


**Figure S2** Confocal microscopy analyses of the lumbosacral spinal cord at peak disease. The left panel shows representative images of immunofluorescence co-localization staining of NLRP3, ASC, caspase-1, or GSDMD in the lumbosacral spinal cord of WT and IRAK-M^-/-^ EAE mice. Scale bar: 50 μm. The corresponding charts show the mean fluorescence intensity (MFI) of NLRP3^+^, ASC^+^, Caspase-1^+^, and GSDMD^+^ inflammatory cells in the lumbosacral spinal cord from mice sacrificed at peak disease (n = 6 per group). Data are shown as the mean ± SEM, ^***^*P* < 0.001, versus the indicated group.

Supplementary Figure 3


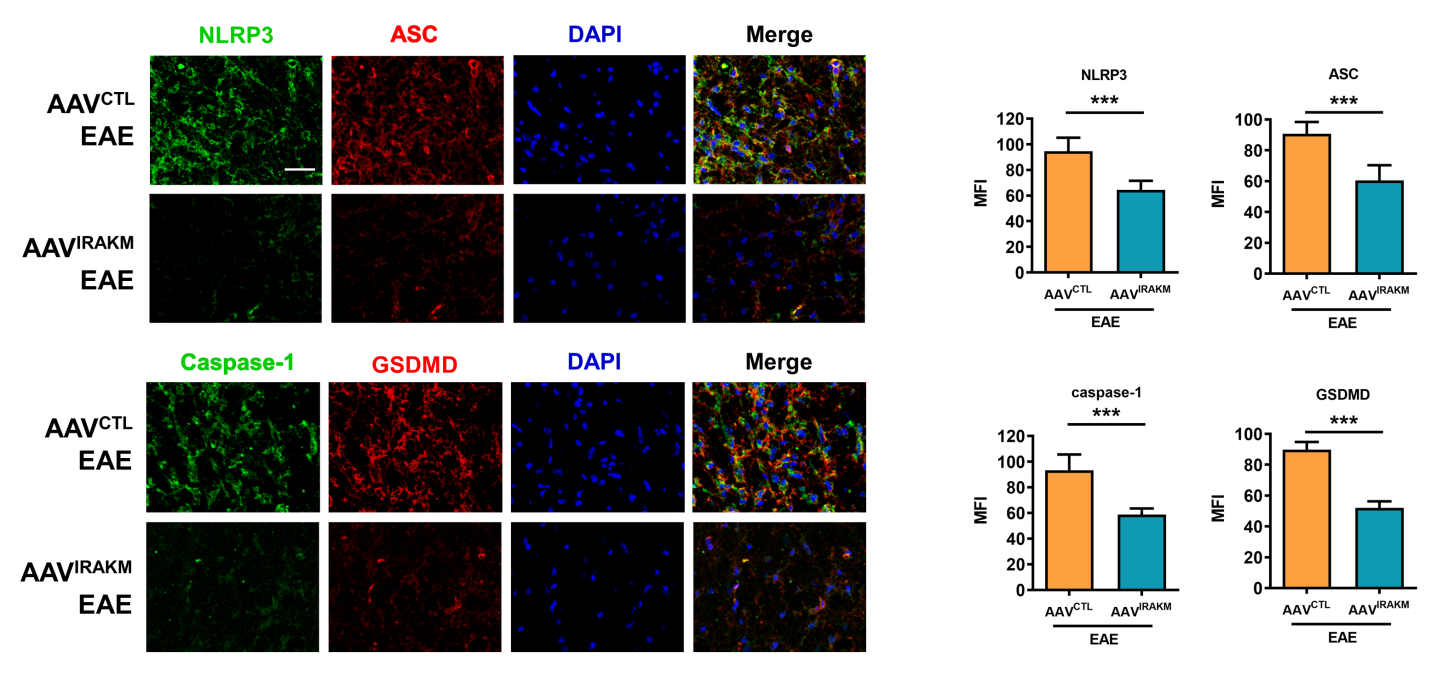


**Figure S3** Confocal microscopy analysis of the lumbosacral spinal cord. The left panel shows representative images of immunofluorescence co-localization staining of NLRP3, ASC, caspase-1, and GSDMD in the lumbosacral spinal cord of AAV^IRAK-M^ and AAV^CTL^ mice. Scale bar: 50 μm. The corresponding charts show the mean fluorescence intensity (MFI) of NLRP3^+^, ASC^+^, Caspase-1^+^, and GSDMD^+^ inflammatory cells in the lumbosacral spinal cord from mice sacrificed at peak disease (n = 6 per group). Data are shown as the mean ± SEM, ^***^*P* < 0.001, versus the indicated group.
