# Supplementary material for: IRAK-M suppresses the activation of microglial NLRP3 inflammasome and GSDMD-mediated pyroptosis through inhibiting IRAK1 phosphorylation during experimental autoimmune encephalomyelitis
Source: Cell Death Dis. 2023 Feb 10;14(2):103. doi: 10.1038/s41419-023-05621-6 (PMC9918485; doi:10.1038/s41419-023-05621-6)
Supplement: Supplementary file 4 — Original Data File [file 41419_2023_5621_MOESM4_ESM.pdf]

Source data for Figure 1A

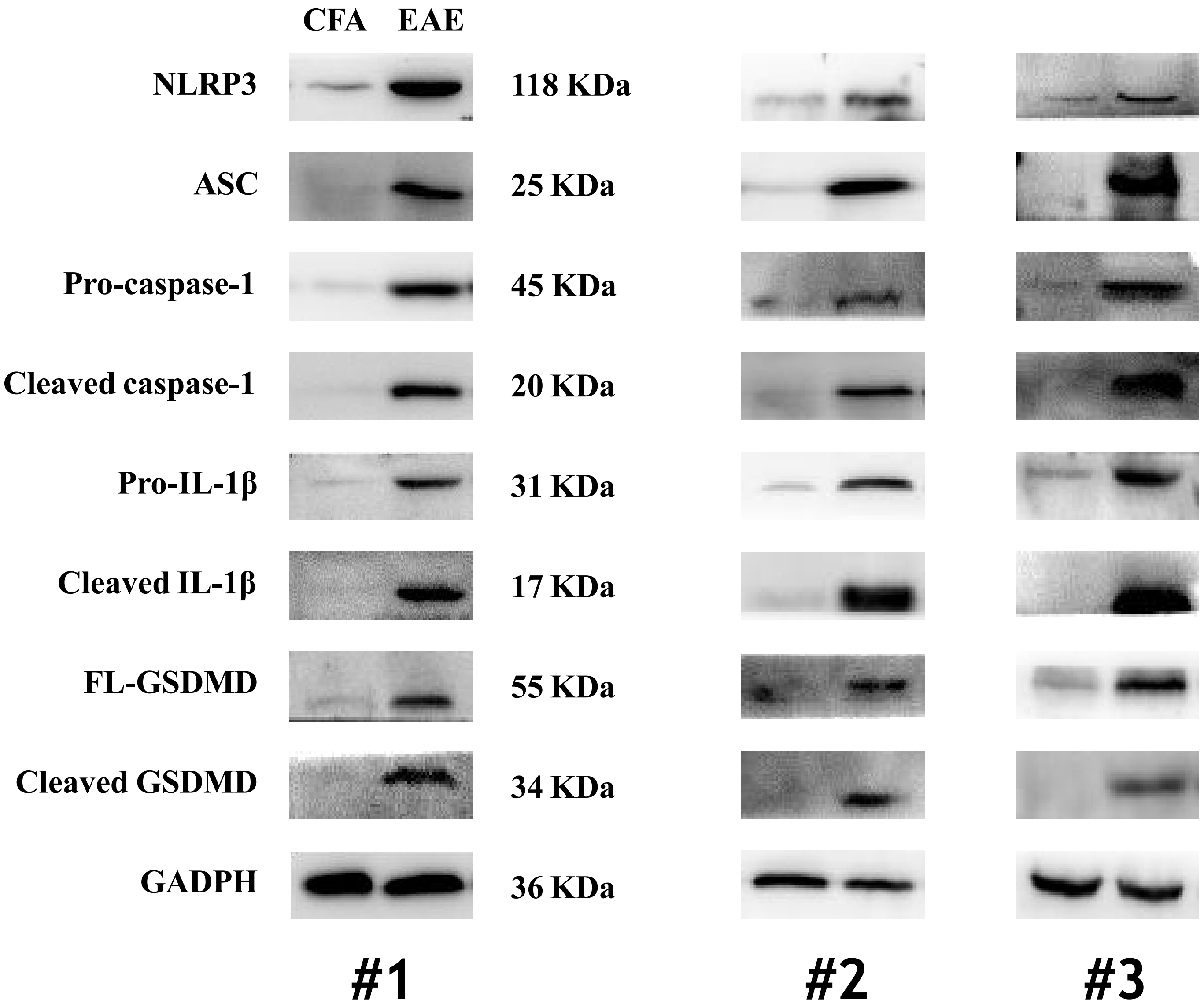

Source data 1. The protein bands of western blotting experiments. n = 3, bands in the #1 were used as representative images in the article.

Source data for Figure 3C

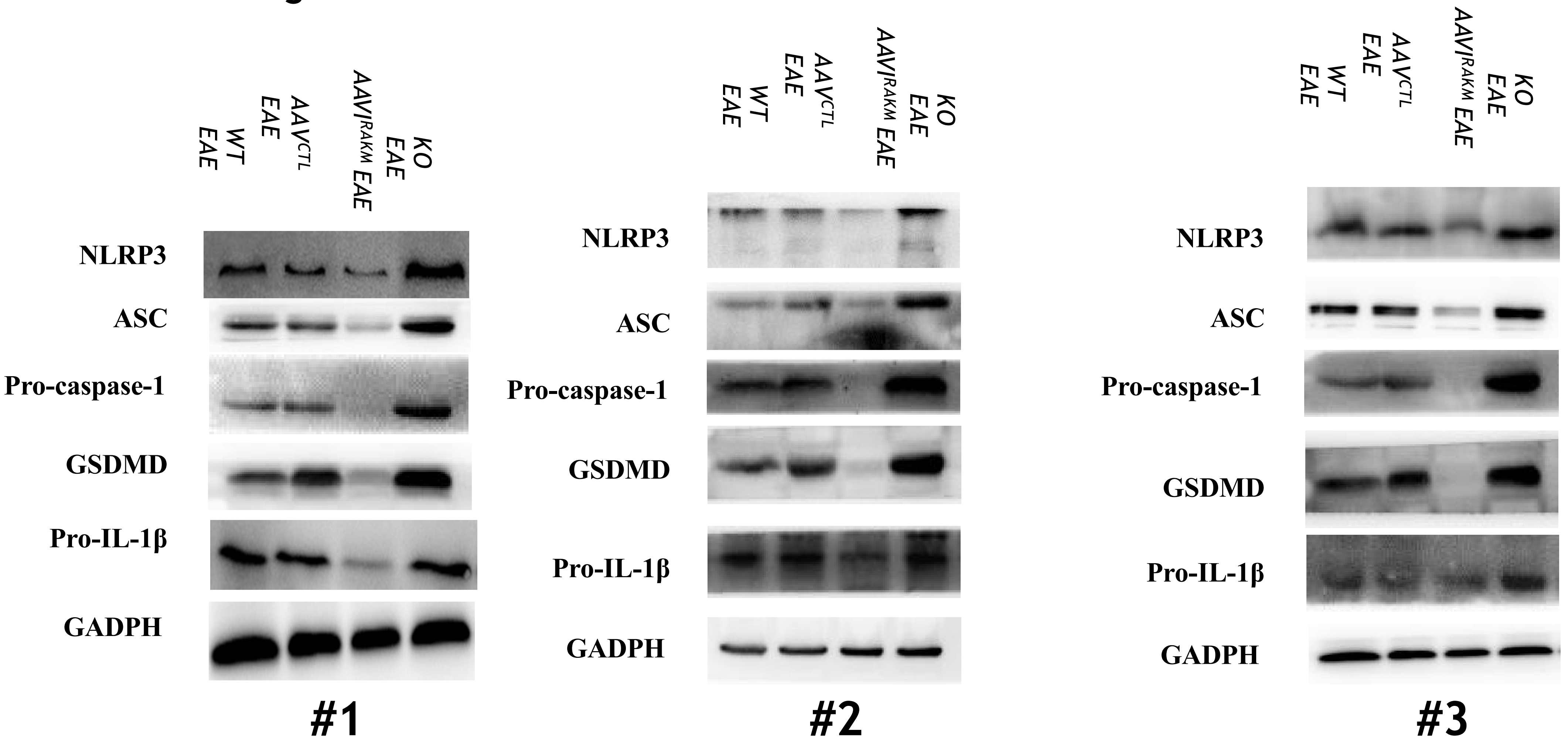

Source data 2. The protein bands of western blotting experiments. n = 3, bands in the #1 were used as representative images in the article.

Source data for Figure 4A

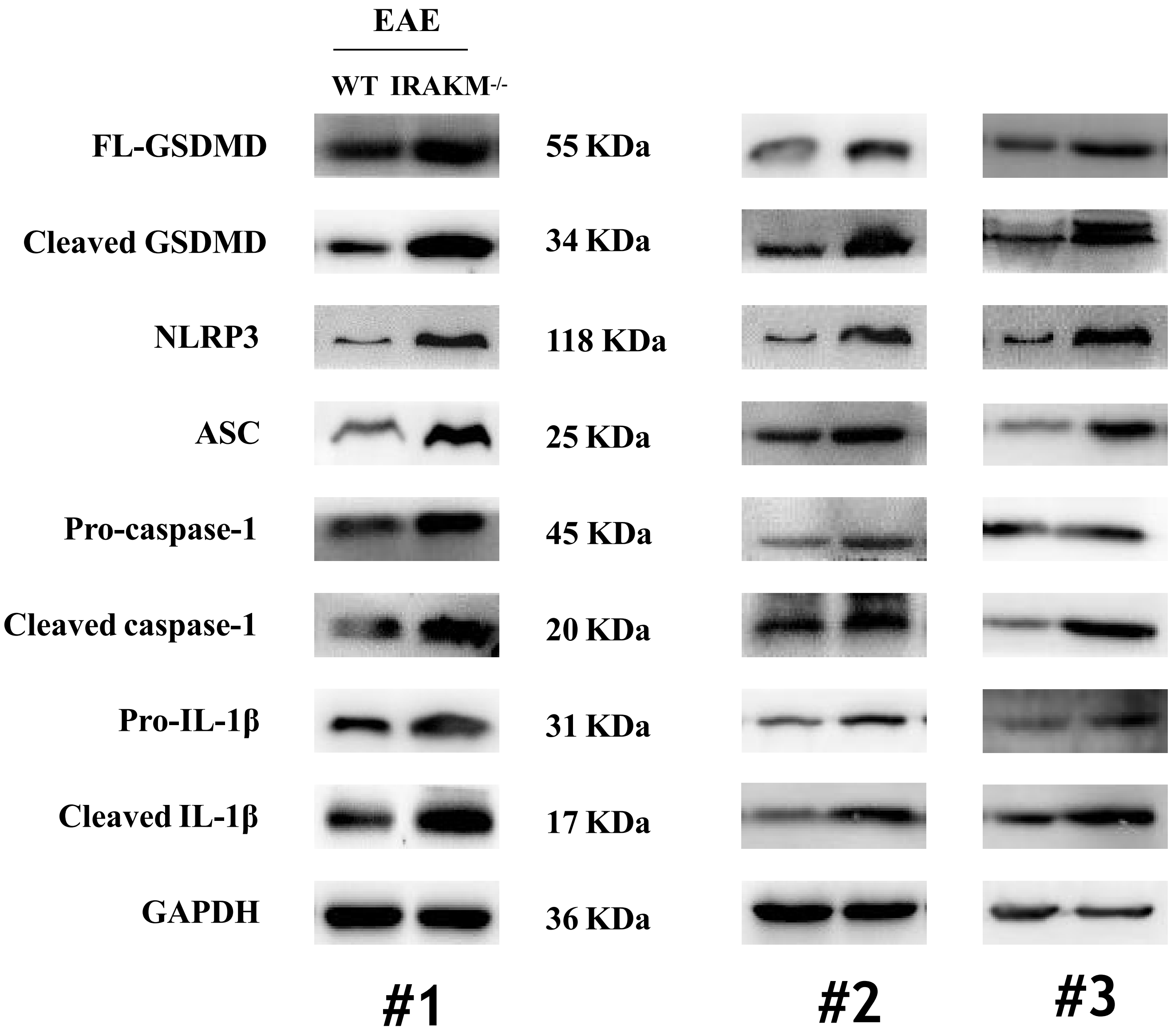

Source data 3. The protein bands of western blotting experiments. n = 3, bands in the #1 were used as representative images in the article.

Source data for Figure 6B

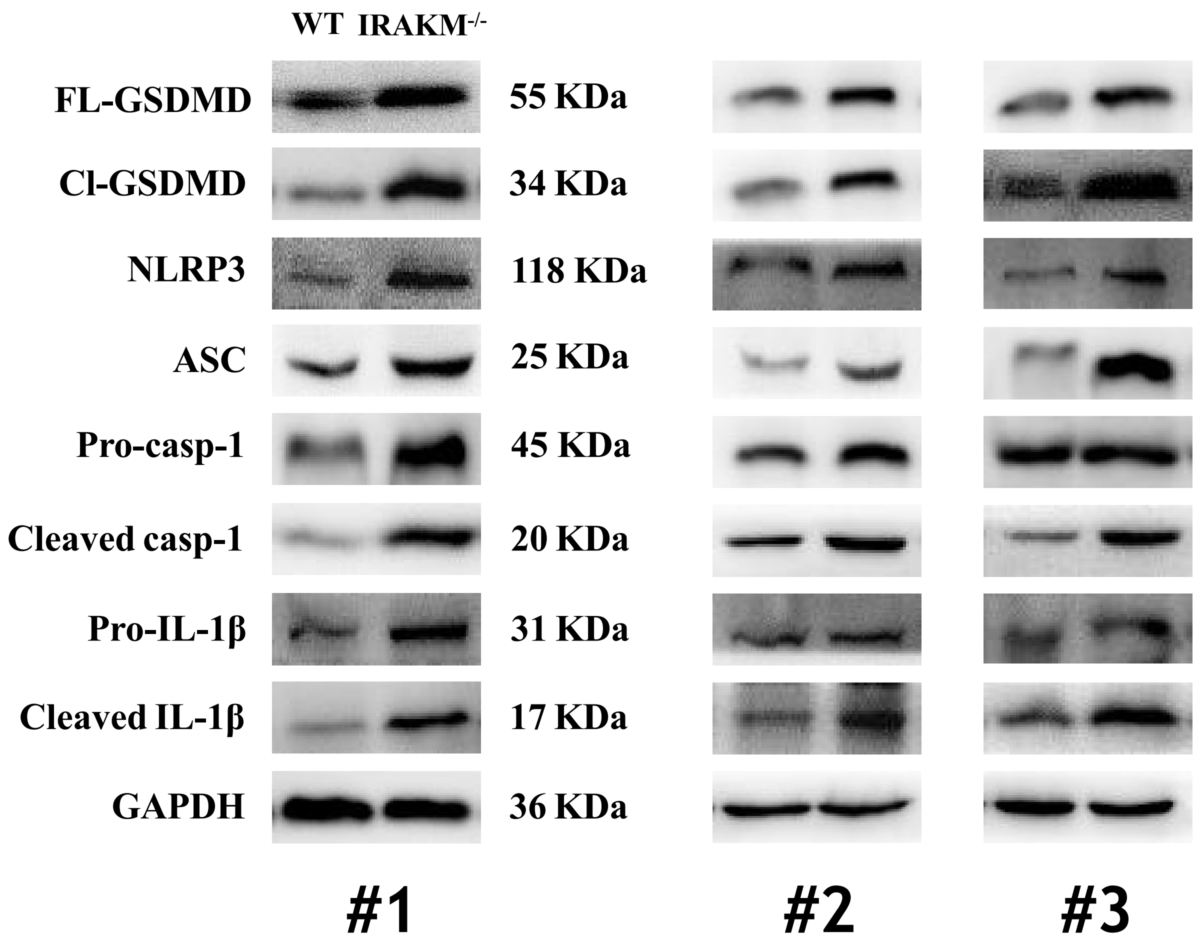

Source data 4. The protein bands of western blotting experiments. n = 3, bands in the #1 were used as representative images in the article.

Source data for Figure 7A

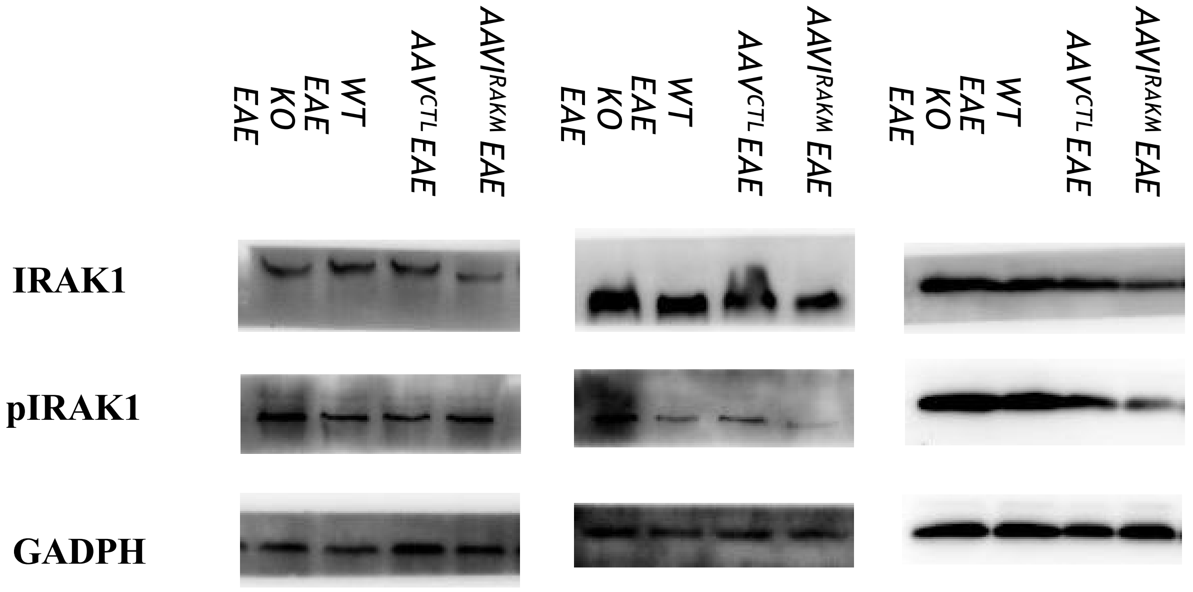

Source data for Figure 7B

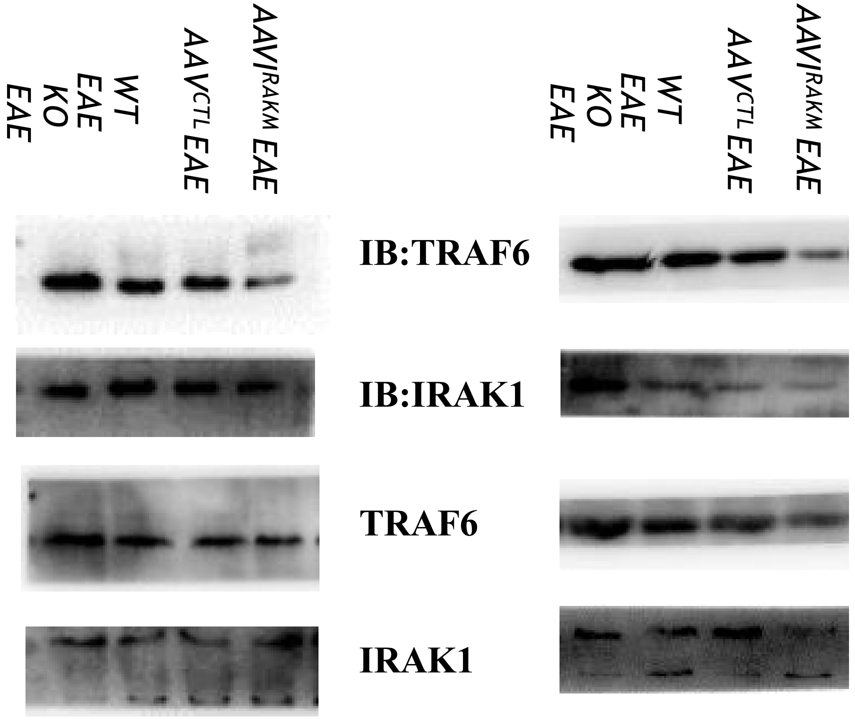

Source data 5. The protein bands of western blotting experiments. n = 3, bands in the #1 were used as representative images in the article.

Source data for response Figure 3A

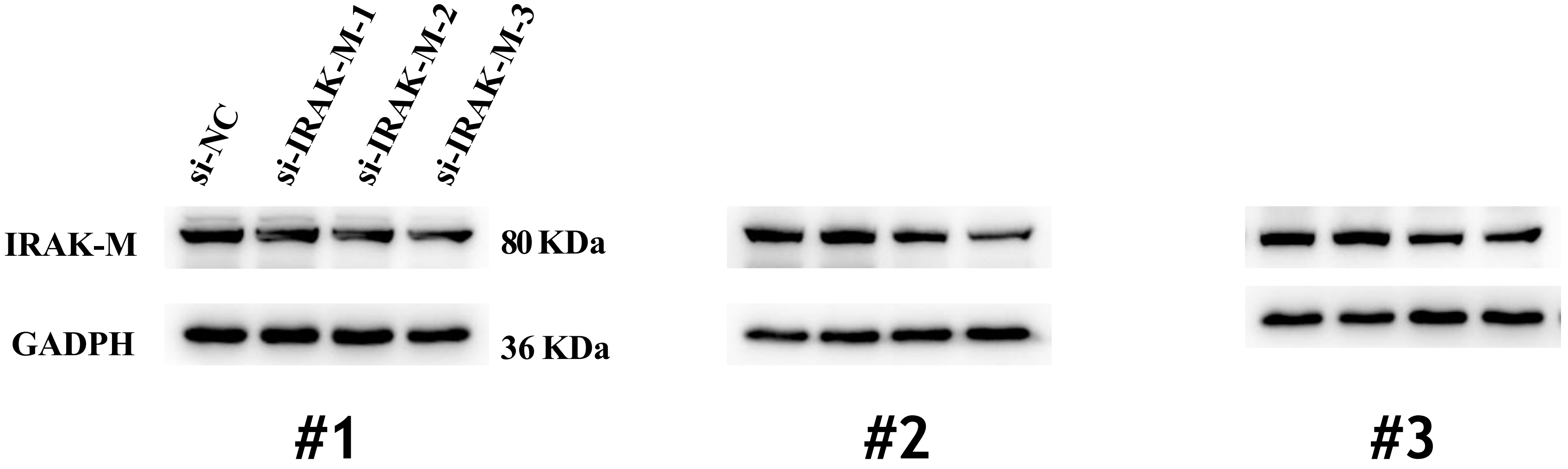

Source data 6. The protein bands of western blotting experiments. n = 3, bands in the #1 were used as representative images in the article.

Source data for response Figure 3C

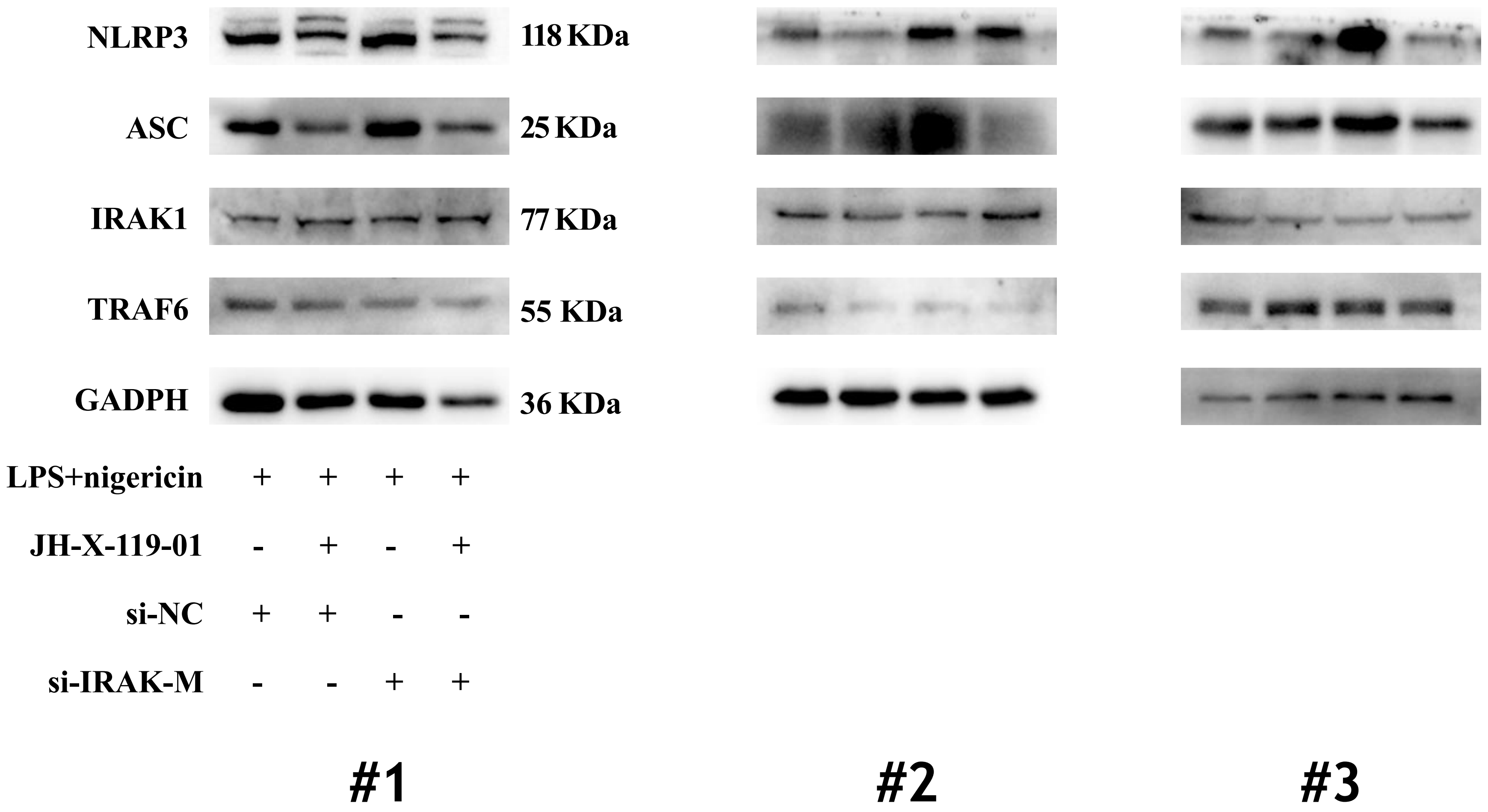

Source data 7. The protein bands of western blotting experiments. n = 3, bands in the #1 were used as representative images in the article.

Source data for response Figure 4A

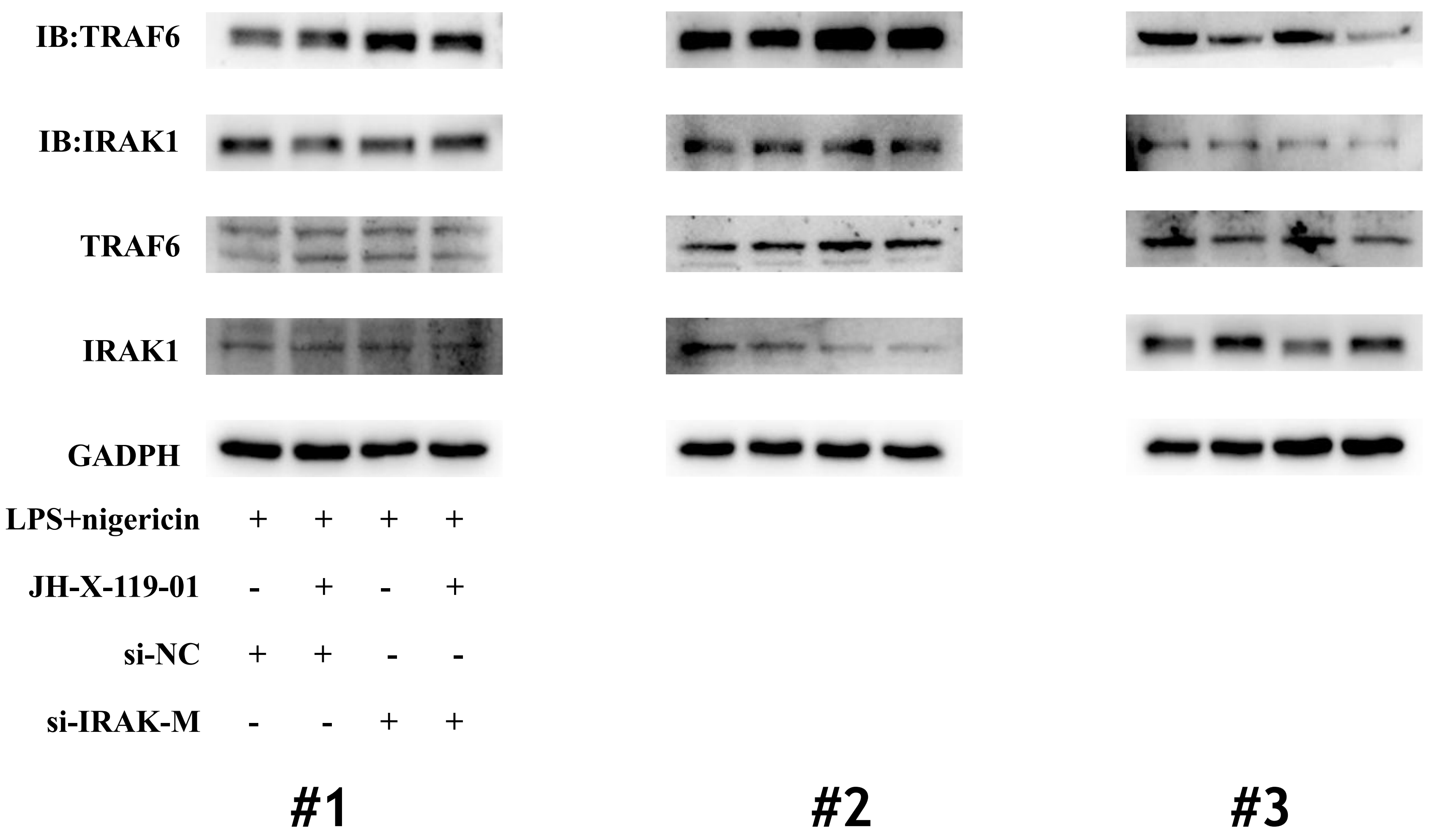

Source data 8. The protein bands of western blotting experiments. n = 3, bands in the #1 were used as representative images in the article.
